# Potent and selective chemical probe of hypoxic signalling downstream of HIF-α hydroxylation via VHL inhibition

**DOI:** 10.1038/ncomms13312

**Published:** 2016-11-04

**Authors:** Julianty Frost, Carles Galdeano, Pedro Soares, Morgan S. Gadd, Katarzyna M. Grzes, Lucy Ellis, Ola Epemolu, Satoko Shimamura, Marcus Bantscheff, Paola Grandi, Kevin D. Read, Doreen A. Cantrell, Sonia Rocha, Alessio Ciulli

**Affiliations:** 1 Division of Biological Chemistry and Drug Discovery, School of Life Sciences, University of Dundee, Dow Street, Dundee DD1 5EH, Scotland, UK; 2 Division of Cell Signaling and Immunology, School of Life Sciences, University of Dundee, Dow Street, Dundee DD1 5EH, Scotland, UK; 3 Cellzome GmbH, Meyerhofstrasse 1, 69117 Heidelberg, Germany; 4 Center for Gene Regulation and Expression, School of Life Sciences, University of Dundee, Dow Street, Dundee DD1 5EH, Scotland, UK

## Abstract

Chemical strategies to using small molecules to stimulate hypoxia inducible factors (HIFs) activity and trigger a hypoxic response under normoxic conditions, such as iron chelators and inhibitors of prolyl hydroxylase domain (PHD) enzymes, have broad-spectrum activities and off-target effects. Here we disclose VH298, a potent VHL inhibitor that stabilizes HIF-α and elicits a hypoxic response via a different mechanism, that is the blockade of the VHL:HIF-α protein–protein interaction downstream of HIF-α hydroxylation by PHD enzymes. We show that VH298 engages with high affinity and specificity with VHL as its only major cellular target, leading to selective on-target accumulation of hydroxylated HIF-α in a concentration- and time-dependent fashion in different cell lines, with subsequent upregulation of HIF-target genes at both mRNA and protein levels. VH298 represents a high-quality chemical probe of the HIF signalling cascade and an attractive starting point to the development of potential new therapeutics targeting hypoxia signalling.

## Abstract (web summary)

Small molecule probes used to trigger hypoxic response by activating hypoxia inducible factors (HIFs) often lack specificity. Here the authors report a potent small molecule inhibitor that induces hypoxic response by blocking VHL:HIF interactions, providing a selective route to probe hypoxic signalling.

## Graphical abstract

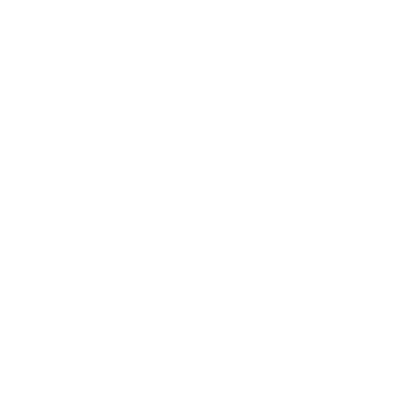

Small molecules that bind potently and selectively to their protein target inside the cell can be high-value chemical tools, complementary to genetic approaches, to study and perturb biological systems. In recent years, development of high-quality chemical probes has enabled significant progress in both basic and translational research, and has furnished suitable start points for drug development^1^,^2^. One area that is seeing increasing interest for chemical biology and drug discovery applications is the pharmacological modulation of oxygen sensing^3^,^4^, one of the most fundamental processes in aerobic organisms^5^. Studying the cell's adaptive responses to low oxygen levels via the hypoxic signalling pathway has provided important insights to aid understanding of oxygen sensing^6^,^7^. Thus the ability to induce hypoxic signalling in a selective fashion using chemical probes with defined mechanism of action could lead to new biological insights, the chemical validation of potential drug targets, and the development of new therapeutics.

Hypoxia inducible factors (HIFs), the master regulators of hypoxic signalling, are a family of oxygen-sensitive transcription factors composed of an oxygen-labile α-subunit HIF-α (of which three paralogs are known: HIF-1α, HIF-2α and HIF-3α) and a constitutively stable β-subunit (HIF-β)^7^. HIF-α, among which HIF-1α and HIF-2α are predominant, are maintained at very low levels under normoxia as a result of highly efficient polyubiquitination by the von Hippel–Lindau (VHL) Cullin RING E3 ubiquitin ligase complex (CRL2^VHL^) and subsequent proteasomal degradation^8^,^9^,^10^. Key to the highly specific molecular recognition of HIF-α by VHL is the post-translational hydroxylation of key HIF-α proline residues by the oxygen-dependent activity of prolyl hydroxylase domain (PHD) enzymes^11^,^12^,^13^,^14^. In contrast, under low oxygen levels HIF-α proteins remain unhydroxylated, escape VHL recognition and proteasomal degradation, and form heterodimeric complexes with HIF-β that bind to a specific core DNA motif known as hypoxia response elements (HREs)^15^. Transcriptionally active HIFs induce the expression of a wide range of genes linked to key biological processes including angiogenesis, cell proliferation, glucose uptake and anaerobic metabolism, ultimately promoting a hypoxic response^6^,^16^,^17^.

Several chemical agents stabilize HIF-α and induce a hypoxic response even under normoxic conditions. Examples of hypoxia mimetic agents include Fe^2+^ substitutes (Co^2+^, Ni^2+^)^18^; 2-oxoglutarate mimics such as dimethyloxalylglycine, *N*-oxalylglycine and *N*-oxalyl-D-phenylalanine^19^,^20^; Fe^2+^ chelators (deferoxamine also known as desferrioxamine, and AKB-4924)^18^,^21^; inhibitors of PHD enzymes, for example, IOX2 (ref. 22) and the phase 3 clinical trial compound roxadustat (FG-4592)^23^; inhibitors of cullin neddylation (MLN4924)^24^,^25^; and proteasome inhibitors^26^. Although widely used in biochemical and biomedical research, these reagents have poor target selectivity and affect multiple pathways, severely limiting their scope as chemical probes of hypoxic signalling. Here, we describe VH298, a novel potent chemical probe that triggers the hypoxic response via a different mechanism, that is, by blocking the VHL:HIF-α protein-protein interaction downstream of HIF-α hydroxylation. We show that VH298 engages with VHL as its only major target to stabilize highly selectively the hydroxylated form of HIF-α in a concentration- and time-dependent fashion in both cancerous and non-cancerous primary cells, with subsequent upregulation of HIF-target genes at mRNA and protein levels.

## Results

### VH298 is a potent inhibitor of the VHL:HIF-α interaction

**Figure 1:**
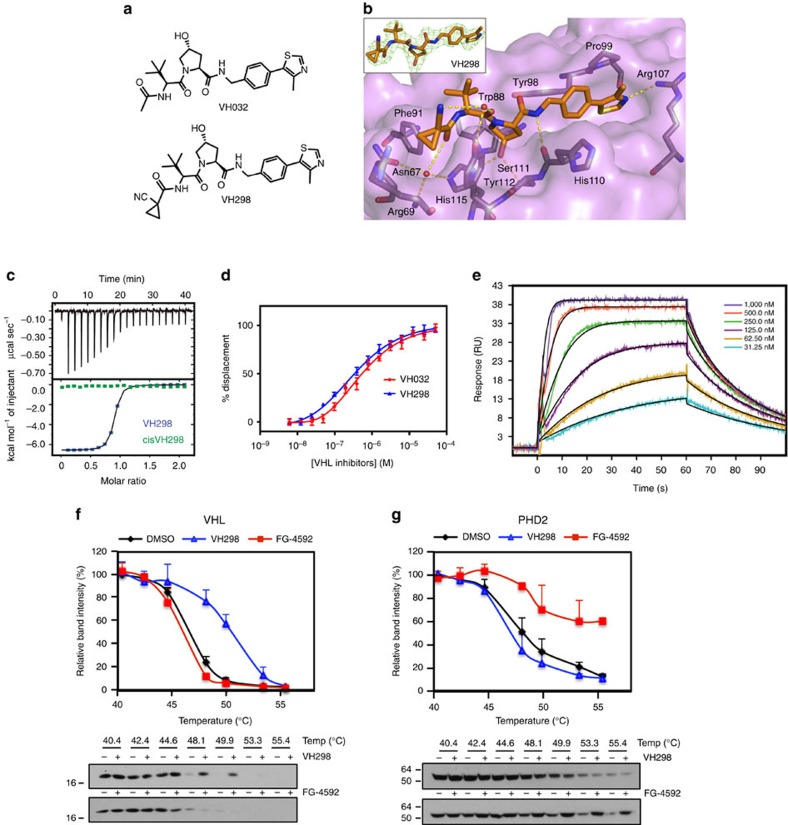
Biophysical and structural characterization of VH298, a new potent VHL inhibitor. (**a**) Chemical structure of VH032 and VH298 inhibitors. (**b**) Crystal structure of VBC in complex with VH298 (orange carbons, PDB code 5LLI). VHL is shown as a purple surface and the VHL residues forming the binding pocket as grey stick representations. The bound ligand is shown as sticks representation with orange carbons, nitrogen atoms in blue, oxygen in red and sulfur in dark yellow. The inset panel shows the Fo-Fc omit map contoured at 3 σ around the ligand. (**c**) ITC titrations of 300 μM VH298 (blue) and 300 μM *cis*VH298 (green) into 30 μM VBC protein. (**d**) Competitive fluorescence polarization binding assays of VH298 and VH032 displacing a 20-mer FAM-labelled HIF-1α peptide binding to VBC (*K*_d_=3 nM), confirming the trend in relative potency observed by ITC (see full data in Table 1). (**e**) SPR sensogram of VH298 binding into surface-immobilized VBC. Used ligand concentrations are reported in the inset legend. Cellular thermal shift assays to monitor cellular target engagement of (**f**) VHL and (**g**) PHD2. VH298 and FG-4592 (100 μM) were incubated in HeLa cell lysates for 15–30 min. Representative western blots are shown. Data are presented as means±s.e.m. from three independent experiments.

First-generation small molecule disruptors of the VHL:HIF-α interaction could not induce HIF accumulation inside cells because of their limited potency^27^,^28^,^29^. To achieve compound-induced HIF-α activity and a hypoxic response, inhibitors would have to bind VHL as strongly as possible to be able to compete with its high affinity for the endogenous HIF-α substrates inside cells (Supplementary Fig. 1). We therefore designed VH298, based on our crystal structure of inhibitor VH032 bound to VHL^30^. VH298 bears a cyanocyclopropyl group in place of the terminal methyl group of VH032 (Fig. 1a). VH298 showed a *K*_d_ of 90 nM as determined by isothermal titration calorimetry (ITC; Fig. 1c and Table 1) and 80 nM in a competitive fluorescence polarization assay (Fig. 1d and Table 1), affording the most potent VHL:HIF-α inhibitor reported to date. This high affinity corresponds to a ligand efficiency of 0.26 that is excellent for a protein–protein interaction inhibitor^31^. In addition, the binding kinetics of the new inhibitor were characterized by surface plasmon resonance (Fig. 1e). In a dose-dependent real-time assay VH298 showed a *k*_on_ of 6.47 × 10^5^ M^−1^ s^−1^ and a *k*_off_ of 0.065 s^−1^, indicating fast association and slow dissociation with VHL complex, respectively (Table 1). To generate a suitable negative control compound that does not bind to VHL but maintains as closely as possible the physicochemical properties of the parent binder, the *cis*-hydroxyproline analogue *cis*VH298 was synthesized (Table 1). This change in stereochemistry at the carbon atom bearing the hydroxyl group of the hydroxyproline ring is known to result in marked loss of binding specificity of hydroxylated HIF-1α peptides to VHL *in vitro*^32^. As expected, we could not detect binding of *cis*VH298 using ITC (Fig. 1c), confirming the stereospecific effect is retained in the context of VHL inhibitors.

### Crystal structure of VH298 bound to its target protein VHL

The crystal structure of VH298 bound to the VHL:ElonginB:ElonginC (VBC) complex (Fig. 1b, stereo view in Supplementary Fig. 2) was solved to 2.4 Å resolution (X-ray data collection, processing and refinement statistics are in Supplementary Table 1). VH298 retains the same overall binding mode of VH032 (ref. 30). The cyanocyclopropyl group fits snugly at the far left-hand side pocket of the HIF-1α binding site on VHL, with the cyclopropyl moiety projecting towards the aliphatic side chain of Arg69, and the cyano group pointing away from the surface and towards solvent in an *anti* conformation relative to the amide carbonyl. This corresponds to a highly favored, minimum energy conformation for amides substituted with electron withdrawing groups at the alpha carbon next to the carbonyl, as well documented for alpha-fluoroamides^33^,^34^. This stabilization would preorganize the ligand in its bound conformation, imparting a boost in binding affinity. Interestingly, Arg69 adopts a bent conformation as opposed to the extended conformation observed in the VH032 bound structure (Supplementary Fig. 3)^30^. This bent conformation of Arg69 has been previously observed when a Leu or *tert*-Leu group of the inhibitor is occupying the far left-hand side pocket^30^, as well as with a bound HIF-1α peptide which bears a Leu residue at this position^13^,^14^, suggesting flexibility of Arg69 could be exploited to accommodate a range of different functional groups at this left-hand side.

### VH298 selectively engages VHL as its prime cellular target

We next asked whether VH298 could interact with cellular VHL protein by performing a cellular thermal shift assay^35^. Protein levels of both on-target VHL and potential off-target PHD2 were monitored at different temperatures of incubation in HeLa cell lysates treated with VH298 using immunoblotting. PHD inhibitor FG-4592 was used as a control in the assay for PHD2 stabilization. In the presence of VH298, a significant shift in the VHL melting curve was observed, demonstrating compound-induced target stabilization inside cells (Fig. 1f). In contrast, no thermal stabilization was observed for PHD2 on VH298 treatment (Fig. 1g). A shift in the melting curve of PHD2, but not VHL, in the presence of FG-4592 (Fig. 1f–g) confirmed the validity of the assay. Target engagement and selectivity of VHL inhibitors to VHL was further supported by chemoproteomic mass spectrometric results. Quantitative mass-spectrometry analysis of proteins using isobaric mass tags^36^ revealed that VHL and other expected CRL2^VHL^ subunits were captured onto beads derivatized with a linkable VH298 analogue and selectively displaced by the active inhibitor (Supplementary Fig. 4 and Supplementary Data 1), but not by an inactive analogue (Supplementary Fig. 5). The results were consistent with those obtained with beads derivatized with hydroxylated HIF-1α oxygen-degradation domain (ODD) peptides (Supplementary Fig. 6 and Supplementary Data 2). Although many proteins were identified in pull-downs with the derivatized resin only the bead binding of components of the CRL2^VHL^ complex was inhibited with increasing concentrations of VHL inhibitor (Supplementary Fig. 4 and Supplementary Fig. 7). We also found VH298 to exhibit negligible off-target effects *in vitro* against >100 tested cellular kinases, GPCRs and ion channels, at 50 μM concentration (Supplementary Table 2 and Supplementary Table 3). Altogether, the biophysical, structural and cell-based *in vitro* data obtained are consistent with a selective high-affinity engagement of VH298 with VHL as its main target.

### VH298 is cell permeable and not toxic to cells

To evaluate the cellular activity of VH298, we first assessed its cell permeability by performing PAMPA (parallel artificial membrane permeability assay) using a filter plate pre-coated with structured layers of phospholipids. We also measured the compounds' lipophilicity at physiological pH (Log*D*_7.4_) as this property is known to directly impact on membrane passive permeability^37^. Higher permeability promotes an increase of available compound inside cells able to interact with the desired target. The measured permeability of VH298 was found to be 19.4 nm s^−1^ , which is acceptable compared with that of high-permeability drug propranolol (63 nm s^−1^, Table 1). Crucially, the permeability of VH298 was significantly higher than that of VH032 (Table 1), in line with the increase in Log*D*_7.4_, suggesting that VH298 can move through cell membranes more efficiently, leading to an increase on available inhibitor in cells. We next examined potential cytotoxicity of VH298 and VH032 in a range of fibroblast, tumoral and non-tumoral cells. The inactive cis epimers of the two VHL inhibitors were also tested to assess whether potential inhibitor toxicity might be due to VHL inhibition or an off-target effect. Pleasingly, the viability of all cell lines tested was unaffected by compound treatments up to 150 μM concentration, and in most cases even up to 500 μM (Supplementary Fig. 8). The high cell permeability and negligible cytotoxicity of VH298 affirmed its potential as chemical probe and warranted evaluation of its biological activity next.

### VH298 leads to HIF-α accumulation inside cells

**Figure 2:**
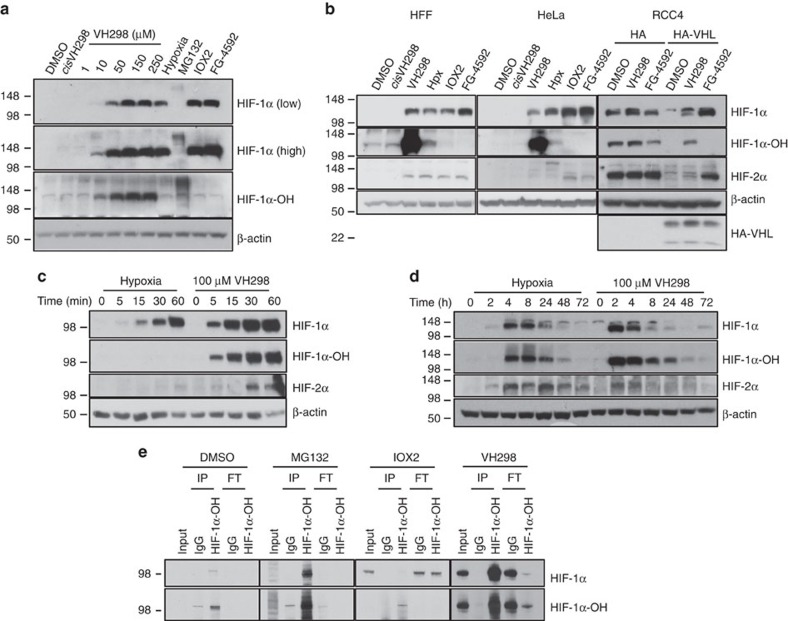
VH298 induces concentration- and time-dependent on-target specific accumulation of hydroxylated HIF-α in human cell lines. (**a**) Immunoblots of HIF-α subunits in HeLa cells treated with increasing concentrations of VH298, 1% DMSO, hypoxia, 250 μM *cis*VH298, 100 μM IOX2, 100 μM FG-4592 for 2 h, and 20 μM MG132 for 3 h. (**b**) Treatment of 1% DMSO, hypoxia (1% O_2_), and 100 μM of indicated compounds in HFF, HeLa, RCC4-HA and RCC4-HA-VHL cells for 2 h. (**c** and **d**) Time-course immunoblots of lysates from HeLa cells subjected to hypoxia (1% O_2_) or 100 μM VH298. (**e**) Co-immunoprecipitation experiments on lysates from HeLa cells treated with vehicle DMSO (1% for 2 h), MG132 (20 μM for 3 h), IOX2 (250 μM for 6 h), or VH298 (100 μM for 2 h) before lysis. 300 μg of protein were used to immunoprecipitate with the HIF-1α-OH (Pro564) antibody. Rabbit immunoglobulin G (IgG) was used as a control. Inputs represent 10% of the starting material used per immunoprecipitation (IP) and flow through (FT) represents 20% of the flow through collected after IP. The blots shown are representative of three independent experiments.

We next asked whether VH298 could stabilize HIF-α inside. VH298 induced concentration-dependent accumulation of HIF-1α levels after 2 h treatments in immortalized HeLa cancer cells (Fig. 2a), with detectable HIF-1α bands visible as low as 10 μM concentration (Fig. 2a). *cis*VH298 was inactive even at high concentration (250 μM), demonstrating that the inhibitor's activity is strictly dependent on binding to VHL. Beside HIF-1α, HIF-2α was also stabilized in the presence of VH298 (Fig. 2b). The extent of VH298-induced HIF-α subunit accumulation after 2 h was comparable to that observed on treating cells over the same time with 1% O_2_ (hypoxia control) or PHD2 inhibitors (IOX2 or FG-4592) at identical concentrations (100 μM) in all cell lines tested (Fig. 2a–b and Supplementary Fig. 9). Immunoblots with antibodies specific for HIF-1α hydroxylated at Pro564 (HIF-1α-OH) showed increased levels of this species only on treatment with VH298 (Fig. 2a–b) and proteasome inhibitor MG132 (Fig. 2a and Supplementary Fig. 10), but not with IOX2 and FG–4592 (Fig. 2a–b and Supplementary Fig. 10), consistent with disruption of the VHL:HIF-α interaction by VH298 downstream of HIF-α hydroxylation. The same cellular profile was observed in the presence of two different VHL inhibitors, VH032 (*K*_d_=185 nM^30^, Fig. 1a) and VH125 (*K*_d_=280 nM^30^; Supplementary Fig. 4) but not with inactive epimers (Supplementary Fig. 10), providing additional evidence for the compounds' mechanism of action.

To further evaluate the activity of VHL inhibitors, time-course experiments with HeLa cells under treatment of VH298 and hypoxia (1% O_2_) were performed and levels of HIF-1α and HIF-2α were independently monitored by immunoblots. VH298 induced a more rapid and pronounced accumulation of HIF-1α and HIF-2α compared with hypoxia (Fig. 2c) and a long-lasting buildup of both HIF-1α and HIF-2α (Fig. 2d) in a time-dependent fashion, with protein levels detectable already after 5 min and persisting beyond 24 h after treatment. To confirm that the increase of HIF-α was indeed due to the inhibition of VHL activity, clear cell renal cell carcinoma *VHL*^−/−^ renal cell carcinoma 4 (RCC4) cells were treated with VH298. In the complete absence of functional VHL proteins in RCC4 cells expressing only the haemagglutinin (HA) tag, the levels of HIF-α subunits did not increase further on VH298 treatment (Fig. 2b). In contrast, RCC4 cells expressing HA-tagged VHL showed accumulation of HIF-α subunits in the presence of VH298 (Fig. 2b and Supplementary Fig. 9), demonstrating the on-target specificity of VH298. Taken together, these data provide the first evidence for the stabilization of HIF-α by VHL inhibitors in tumoral and non-tumoral cell lines, resulting from effective and specific blockade of the VHL:HIF-α interaction.

### VH298 selectively stabilizes hydroxylated HIF-α

To further interrogate the specificity of the compound's mode of action, we next asked whether all the stabilized HIF-1α is hydroxylated on treatment with VH298. Since the HIF-1α antibody does not discriminate between hydroxylated and non-hydroxylated HIF-1α species, while the HIF-1α-OH antibody is specific for the hydroxylated species (Fig. 2a–b), we performed immunoprecipitation experiments with the HIF-1α-OH specific antibody. Immunoblotting of HIF-1α after treating cells with VH298 showed that HIF-1α was found in the immunoprecipitation fractions and not in the flow-through, consistent with the HIF-1α species stabilized by VH298 being all of the hydroxylated form (Fig. 2e). The same result was found when we performed an identical experiment with the proteasome inhibitor MG132, which also blocks the pathway downstream of HIF hydroxylation. In contrast, when cells were treated with the upstream PHD inhibitor IOX2, HIF-1α was found in the flow-through and not in the immunoprecipitation fractions, consistent with the HIF-1α stabilized by IOX2 instead being unhydroxylated (Fig. 2e). Together these results are consistent with VH298 blocking VHL E3 ubiquitin ligase downstream of HIF hydroxylation, leading to specific intracellular accumulation of the hydroxylated HIF-α form.

### VH298 induces HIF transcriptional activity

**Figure 3:**
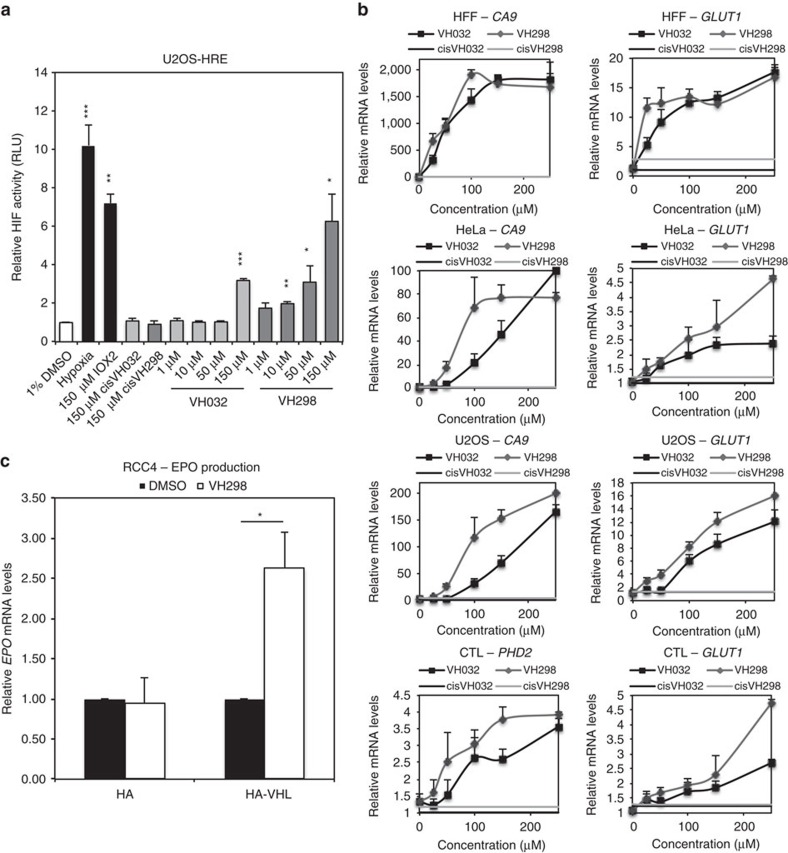
VHL inhibitors induce HIF-α transcriptional activity in various cell lines. (**a**) U2OS cells stably expressing an HRE-luciferase reporter plasmid were treated with indicated conditions for 32 h. (**b**) Dose-response curves of *CA9* and *GLUT1* mRNA expressions in HFF (24 h), HeLa (16 h), U2OS (16 h) and CTL (6 h) cells in the presence of *cis*VH032, *cis*VH298, VH032 and VH298. mRNA was collected, reverse transcribed and analysed by qRT-PCR. The shown levels of the indicated mRNAs were normalized to those of β-actin, except for CTLs where they were normalized to the levels of TATA-binding protein mRNA. (**c**) EPO production in RCC4-HA and RCC4-HA-VHL cells subjected to 150 μM VH298. Graphs depict the mean+s.e.m. of three independent biological replicates. Student's *t*-test was performed to calculate *P* values, and levels of significance are denoted as follows: *P<0.05, **P<0.01 and, ***P<0.001.

To assess the ability of VHL inhibitors to promote HIF activity, we first used a luciferase reporter assay^38^. In HRE-luciferase reporter cells, U2OS-HRE, treatments with VHL inhibitors exhibited marked concentration-dependent increase of HIF-dependent luciferase activity, consistent with hypoxia and IOX2 treatments, while no activity was observed with high concentration of non-binding epimers (Fig. 3a). Corresponding to VH298 being more potent and more cell-permeable than VH032 (Table 1), VH298 required a lower concentration (50 μM) than VH032 (150 μM) to reach the same level (threefold increase relative to control) of HIF activity in U2OS cells (Fig. 3a). VH298 induced detectable HIF activity at 10 μM concentration (Fig. 3a). The more potent cellular activity of VH298 compared with VH032 was further confirmed in a qRT–PCR assay (Fig. 3b) monitoring mRNA levels of known HIF-target genes including *CA9*, *GLUT1* and *PHD2* in different cellular backgrounds. Treatments with VH032 and VH298 showed marked upregulation of HIF-target genes, but not with inactive epimers *cis-*VH032 and *cis-*VH298 (Fig. 3b). Different responses to VHL inhibitor treatment were observed in different cell lines. For instance, HFF was the most sensitive to VHL inhibitors among the cell lines used; maximum mRNA levels of *CA9* and *GLUT1* were reached with 100 and 25 μM of VH298, respectively, in HFF cells. Consistently in all cell lines, a lower concentration of VH298 than VH032 was required to increase mRNA levels of target genes to the same level. These results indicate that VHL inhibitor-induced hydroxylated HIF proteins are transcriptionally active and confirm the superiority of VH298 over previously reported VHL inhibitors as a chemical probe.

### VH298 stimulates EPO production in a VHL-dependent manner

HIF transcriptional activities are known to stimulate the production of the hormone erythropoietin (EPO), which is primarily expressed in the kidney and liver^39^. To investigate whether VH298-induced HIF activity is able to stimulate EPO production, *EPO* mRNA levels in RCC4 cells reconstituted with HA-tag or HA-VHL tag were analysed by qRT–PCR. VH298 increased mRNA levels of *EPO* by 2.5-fold in RCC4-HA-VHL, but not in VHL-null RCC4-HA (Fig. 3c), indicating that pharmacological inhibition of VHL is able to stimulate endogenous EPO synthesis.

### VH298 upregulates HIF-dependent response gene products

**Figure 4:**
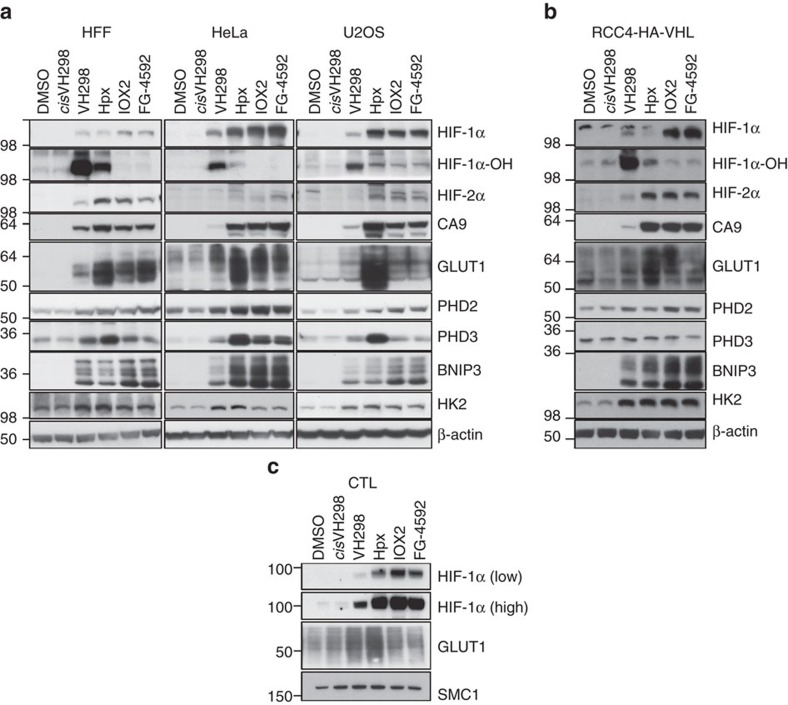
VH298 elicits a HIF-dependent hypoxic response in various cell lines. 1% DMSO, hypoxia (1% O_2_) and indicated compounds at 100 μM were introduced to (**a**) HFF, HeLa, U2OS and (**b**) RCC4 cells expressing HA-tagged VHL (with the exception of *cis-*VH298 and VH298 used at 150 μM) for 24 h and (**c**) CTLs for 8 h. Protein levels were analysed by immunoblotting using antibodies against the indicated proteins, with β-actin (SMC1 for CTLs) as the loading control. The blots shown are representative of three independent experiments.

To examine whether the observed VH298-dependent increases of HIF target mRNAs would be translated into proteins, we monitored protein levels of known HIF target gene products (CA9, GLUT1, PHD2, PHD3, BNIP3 (ref. 40) and HK2) in mammalian cells treated for 24 h with VH298 or PHD inhibitors IOX2 and FG-4592 (each at concentration of 100 μM), or hypoxia (1% O_2_). The protein levels of GLUT1 were monitored in cytotoxic T lymphocyte (CTL) cells as *GLUT1* is a known HIF-1α target gene in these cells^41^. Corresponding to the stabilized HIF subunits, accumulation of all the HIF targets was detected following prolonged treatment of VH298 in cells, consistent to hypoxia or PHD inhibition treatments (Fig. 4a–c). In all cell lines tested, many of the HIF targets screened were increased the most in the hypoxia condition, with GLUT1 and CA9 being the most prominent (Fig. 4a–c). PHD3 was also upregulated the most under hypoxia in most cell lines (Fig. 4a,c), but not in RCC4-HA-VHL, in which PHD3 levels were not affected by any treatments (Fig. 4b). VH298 proved as effective as hypoxia in raising PHD2 and HK2 protein levels (Fig. 4a–b), however in HFF the BNIP3 protein level increased more with VH298 treatment than hypoxia treatment. By contrast, most target proteins were upregulated by VH298 to similar levels when compared with PHD inhibition in all cell lines tested (Fig. 4a–b). The exception was RCC4-HA-VHL cells, in which IOX2 and FG-4592 treatments were the most effective conditions, as observed by higher levels of target proteins, including CA9, PHD2, BNIP3 and HK2 (Fig. 4b). Unlike in other cell lines, a higher concentration (150 μM) of VH298 was introduced to RCC4-HA-VHL cells to detect a good response to VHL inhibition, which was likely due to the high level of HA-tagged VHL expressed in the VHL-null RCC4 cells (Supplementary Fig. 11). The data presented support the application of VH298 as chemical probe that elicits effective HIF-dependent hypoxic response inside cells.

## Discussion

Chemical probes that target specific gene products at the post-translational level, rather than at the DNA or RNA level, offer attractive advantages over gene knock-outs and RNA interference; including allowing spatial and temporal control, convenience of delivery, stability and reversibility of action, and the ability to address different post-translational isoforms or modification states. Target inhibition by a chemical probe is also mechanistically different from target knockout or knockdown, and as a result often leads to different phenotypic outcomes. Chemical probes are, therefore, complementary to genetic tools in that they allow addressing biological questions about the molecular target in different ways, often leading to new insights^2^. Genetic knockouts and knockdown to inactivate VHL have been widely used in biochemical research. While homozygous *VHL*^−/−^ mice are embryonically lethal and not viable^42^, heterozygous and conditional tissue-specific *VHL* knockouts^43^, as well as *VHL* small interfering RNA^44^, have provided useful tools shining important insights in the biological consequences of VHL inactivation and subsequent HIF upregulation in cancer^45^, immunology and inflammation^46^, and aging^47^. However, the functional consequences of blocking the VHL:HIF-α interaction with small molecules inside cells had to date remained elusive and limited to early studies using ectopically expressed or *TAT*-fused ODD polypeptide sequences^48^. Here we describe a different approach to HIF stabilization and hypoxic response, through the targeting of VHL using the potent and cell-penetrant small molecule VH298 that disrupts the interaction of VHL with HIF-α. We report systematic profiling of VH298 in cells, not only in terms of specificity of mechanism of inhibition, but also potency, target engagement and exposure, and selectivity. Our data provide strong cellular validation for the application of VHL inhibitors as chemical probes to study specific roles of VHL and hydroxylated HIF-α in biology, which could help to dissect HIF-dependent functions of VHL from HIF-independent functions^49^.

Disruption of VHL function in *VHL* disease mutations of the von Hippel–Lindau syndrome or in *VHL*^−/−^ RCC leads to constitutive stabilization and upregulation of HIF-α even in the absence of hypoxia, driving pro-angiogenic transcription^45^,^50^,^51^. In contrast to the roles of HIF in renal cell carcinoma tumorigenesis, its pharmacological stabilization under normoxic conditions could provide therapeutic benefit for many conditions including anemia due to chronic kidney disease and anemia associated to cancer chemotherapy^52^, ischemia and ischemic reperfusion injuries in the kidney, brain, heart or liver^53^,^54^, acute lung injuries^55^ and intestinal inflammation^56^. Recent studies have also identified VHL knockdown/knockout as protective during states of mitochondrial dysfunction, supporting VHL and PHD inhibition as a potential treatment for human diseases associated with mitochondrial respiratory chain dysfunction^57^. Over the years, a number of strategies have been developed to stabilize HIF-α through escaping or blocking its degradation by the proteasome, with the most promising being the inhibition of HIF-α hydroxylation catalysed by PHDs^58^. Five PHD inhibitors (BAY-853934, JTZ-951, FG-4592, AKB-6548 and GSK1278863) have entered clinical trials, with the most advanced (FG-4592) being in Phase III trials to treat anemia in patients with chronic kidney disease^23^. PHD inhibitors have shown clinical efficacy in Phase II trials, and have largely proved safe, thus validating the hypoxic signalling pathway as a drug target for these conditions^3^. However, side- and off-target effects of PHD inhibitors have raised concerns about their safety and tolerability as therapies for human patients, as exemplified by the failure of early candidate FG-2216 that caused fatal hepatic necrosis^59^. Three PHD isoenzymes are known (PHD1, PHD2 and PHD3) that have different substrate specificities and different subcellular and tissular expression^60^. In addition, other substrates of PHDs have been identified beyond HIF, including Cep192, RNA polymerase and the β-adrenergic receptor^60^,^61^. Potent and highly selective VHL inhibitors represent an attractive alternative to PHD inhibitors as HIF-stabilizing agents with a different mechanism of action, which may alleviate potential HIF-independent undesired effects of PHD inhibitors. In addition, VH298 is also able to stimulate the production of endogenous EPO (Fig. 3c), indicating its potential to benefit patients with anemia resulting from insufficient EPO synthesis^39^. Our data disclosing VHL inhibitors as new chemical probes of hypoxic signalling warrant further lead optimization and investigation of compound bioavailability, stability and efficacy *in vivo* to exploit the full therapeutic potential of this class of compounds in future.

While maximizing HIF-stabilizing activity is desirable for the therapeutic purpose of developing VHL inhibitors in their own right, the opposite holds true in the context of proteolysis targeting chimeras (PROTACs)^62^. PROTACs are heterobifunctional compounds in which a small molecule binding to a Cullin-RING E3 ligase^63^ can be chemically linked to another ligand to induce intracellular degradation of a specific target protein^64^. Recent studies have reported promising biological efficacies in cells and *in vivo* against a range of targets with different PROTACs recruiting the VHL E3 ligase, many using VH032 as the VHL ligand^65^,^66^. In this context, it is important that compounds do not induce a HIF-dependent response, which would otherwise confound or interfere with the desired biological effect resulting from induced target knockdown. The results of this study reveal an exploitable concentration window between the desired PROTAC activity (observed in the pM-nM range^65^,^66^,^67^) and the HIF-stabilizing activity (observed in the μM range with VHL inhibitors, documented here).

In summary, we describe and characterize VH298 as a potent and selective VHL inhibitor that triggers a functional dose-dependent response downstream of HIF-α hydroxylation in the hypoxia-signalling cascade inside cells. This is to our knowledge the first report to date of selective pharmacological stabilization of the hydroxylated form of HIF-α using small molecules chemical probes to drive a HIF-dependent hypoxic response. Relevant information to the use of VH298 as a new chemical probe of the hypoxic signalling pathway will be deposited in the newly established ‘Chemical Probes Portal' (http://www.chemicalprobes.org/)^2^ for the benefit of the scientific community. We anticipate that the data reported in this study and the availability of the compound will aid wide acceptance and use by the community and lead to many new advances in the hypoxia field.

## Methods

### Synthesis of VH298

Synthetic methods are included in the Supplementary Methods of the Supporting Information.

### Isothermal titration calorimetry

ITC experiments were carried in an ITC_200_ micro-calorimeter (GE Healthcare), as previously described^30^.

### Fluorescence polarization

Fluorescence polarization competitive binding experiments were performed on a PHERAstar FS (BMG LABTECH) in 384-well plates (Corning 3575), with λ excitation at 485 nm and λ emission at 520 nm. Each well solution (15 μl) contained 15 nM of VBC protein, 10 nM of 5,6-Carboxyfluorescein (FAM) labelled HIF-1α peptide (FAM-DEALAHypYIPMDDDFQLRSF, *K*_d_=3 nM as measured by a direct fluorescence polarization titration) and decreasing concentrations of compound (13-point serial two-fold dilutions starting from 50 μM), in 100 mM Bis-tris, 100 mM NaCl, 1 mM DTT, pH 7. Control wells contained VBC and peptide in the absence of compound (maximum signal), and peptide in the absence of protein (background signal). Data were obtained in triplicate and fitted using a four-parameter dose-response model using the PHERAstar Mars software provided by the manufacturer. The average IC_50_ and the standard error of the mean (SEM) were determined for each titration. Dissociation constants *K*_d_ were back-calculated from the measured IC_50_ values using a displacement binding model, as previously described^28^.

### Surface plasmon resonance

Compound VH298 was dissolved in dimethylsulfoxide (DMSO) (1 mM) and then diluted 20 fold in DMSO to achieve a 50 μM final stock concentration. Ligand stock solution was diluted twofold (five times) in DMSO and the obtained solutions were then diluted individually in surface plasmon resonance buffer (20 mM HEPES, 150 mM NaCl, 1 mM DDT, 0.005% Tween P20, pH 7.0) to obtain the final 2% DMSO concentration series from 1 μM to 31.25 nM (two-fold dilutions) and transferred to a 96-well plate. The experiment was conducted in a Biacore T100 (GE Healthcare, Biacore, Uppsala, Sweden) at 10 °C and solutions were injected individually using 60 s and 160 s association and dissociation times, respectively. Data were treated using a Biacore T100 Evaluation Software provided by the manufacturer. Reference flow cell response was subtracted from the sample response with immobilized VBC protein to correct for systematic noise and baseline drift. Data were solvent corrected and the response from the blank injections was used to double reference the binding data. The data were molecular weight normalized and *k*_on_/*k*_off_ values were obtained using a 1:1 binding model fit.

### Crystal structure of the VBC:VH298 complex

The VBC ternary complex was purified and crystallized as described previously^28^,^30^. Equal volume solutions of VBC (∼5 mg ml^−l^) and liquor solution were mixed in the hanging-drop vapor diffusion method at 18 °C. The liquor solution contained 0.1 mM sodium cacodylate, pH 6.4, 16% polyethylene glycol 3,350, 0.2 M magnesium acetate and 10 mM DTT. The drop was streaked with seeds of disrupted VBC crystals and a 2–3 mm layer of Al's Oil (Hampton Research) was applied on top of the liquor solution to slow the vapour diffusion rate. To obtain a structure of VH298 bound to VBC, crystals were soaked overnight in a 1 mM solution of inhibitor in 1% DMSO, 4% isopropanol and 95% liquor solution. Crystals were screened using an in-house Rigaku M007HF X-ray generator and Saturn 944HG+ CCD detector. X-ray data were collected at 100 K at Diamond Light Source beamline I04-1. Indexing and integration of reflections was performed using XDS with the XDSGUI interface^68^, and scaling and merging with AIMLESS in CCP4i^69^,^70^. The isomorphous datasets were refined using REFMAC5 (refs 71, 72) and COOT^73^ using a template structure derived from the Protein Data Bank (PDB) entry 1VCB^9^. Ligand structures and restraints were generated using the PRODRG server^74^. The MOLPROBITY server was used to validate the geometry and steric clashes in the structures^75^. The data collection and refinement statistics are presented in Supplementary Table 1.

### Cell culture and hypoxia induction

Human cell lines HFF, HeLa, U2OS, RCC4-HA and RCC4-HA-VHL were obtained from ATCC and propagated in DMEM supplemented with 10% fetal bovine serum (FBS), L-glutamine, 100 μg ml^−l^ of penicillin/streptomycin) at 37 °C. RCC4-HA and RCC4-HA-VHL were maintain in 400 μg ml^−1^ G418. U2OS-HRE luciferase cells were as described^76^ and maintained in 0.1 μg ml^−1^ puromycin. All cell lines were routinely tested for mycoplasma contamination using MycoAlert kit from Lonza. Primary mouse CTLs were generated and cultured as described previously^77^. Briefly, lymphocytes isolated from spleens of nontransgenic mice were activated for 48 h with 0.5 μg ml^−1^ of anti-CD3 (2c11) and 4 ng ml^−1^ of anti-CD28 and 20 ng ml^−1^ of IL-2 (Proleukin) and then maintained in the culture for additional 5 days in 20 ng ml^−1^ of IL-2. For hypoxia induction, cells were incubated at 1% O_2_ in an InVIVO 300 hypoxia workstation (Ruskin Technologies). To prevent reoxygenation, cells were lysed for protein extraction in the hypoxia workstation.

### Immunoblotting

Cells were lysed in RIPA buffer (50 mM Tris pH 8, 150 mM NaCl, 1% NP-40, 0.5% sodium deoxycholate, 0.1% SDS), 250 M Na_3_VO_4_, 10 mM NaF, and a protease inhibitor cocktail (Roche) per 10 ml buffer. CTL cells were lysed at 2 × 10^6^ in Tris lysis buffer containing 10 mM Tris, pH 7.05, 50 mM NaCl, 30 mM Na pyrophosphate, 50 mM NaF, 5 μM ZnCl_2_, 10% glycerol, 0.5% Triton, 1 mM neutralized TCEP and protease inhibitors (Roche). Proteins were resolved using sodium dodecyl sulfate polyacrylamide gel electrophoresis (SDS–PAGE), transferred onto polyvinylidene difluoride membranes and detected using primary antibodies, with β-actin and SMC1 as loading controls in mammalian cells and CTLs, respectively.

Primary antibodies were used at following dilutions for mammalian cells: anti-HIF-1α (BD Biosciences; 610958; 1:1,000), anti-hydroxy-HIF-1α (Hyp564) (Cell Signaling Techonology; #3434; 1:1,000), anti-HIF-2α (R&D; AF 2886; 1:1,000), anti-CA9 (Novus Biologicals; NB100-417; 1:1,000), anti-GLUT-1 (Cell Signaling Techonology; CS 129395; 1:1000), anti-PHD2 (Bethyl Laboratories; A300-322A; 1:1,000), anti-PHD3 (Bethyl Laboratories; A300-327A; 1:1,000), anti-BNIP3 (abcam; ab10433; 1:10,000), anti-VHL (Cell Signaling Techonology; #2738; 1:1,000) anti-β-actin (Cell Signaling Techonology; #3700s; 1:10,000).

Primary antibodies were used at following dilutions for CTLs: anti-HIF-1α (R&D; MAB1536; 1:500), anti-GLUT-1 (Cell Signaling Techonology; CS 129395; 1:1,000) and anti-SMC1 (Bethyl Laboratories, Inc.; A300-055A 1: 10,000).

Following incubation with a horseradish peroxidase-conjugated secondary antibody (Cell Signaling Techonology), chemiluminescence (Thermo Scientific) was used for immunodetection.

### Chemoproteomics

HeLa cells were purchased from ATCC and cultured in MEM supplemented with 1 mM pyruvate, 0.1 mM nonessential amino acids and 10% FCS at 37 °C, 5% CO_2_. Cell pellets were homogenized in lysis buffer (50 mM Tris-HCl, 0.8% Igepal-CA630, 5% glycerol, 150 mM NaCl, 1.5 mM MgCl_2_, 25 mM NaF, 1 mM Na_3_VO_4_, 1 mM DTT, pH 7.5). One complete EDTA-free protease inhibitor tablet (Roche) per 25 ml was added. The sample was dispersed using a Dounce homogenizer, kept rotating for 30 min at 4 °C and spun for 10 min at 20,000 × *g* at 4 °C. The supernatant was spun again for 1 h at 145,000 × *g*. The protein concentration was determined by Bradford assay (BioRad), and aliquots were snap frozen in liquid nitrogen and stored at −80 °C.

For peptide pull-down experiments, biotinylated HIF-1α CODD and NODD 19-mer Hyp-modified peptides (NODD: Hyp402, CODD: Hyp564) or unmodified, harbouring aminohexanoic acid (ahx) as spacer after the biotin (NODD: biotin-ahx-DALTLLA-(Hyp/P)-AAGDTIISLDF-amide; CODD: biotin-ahx-DEALA-(Hyp/P)-YIPMDDDFQLRSF-amide) were immobilized on high capacity Neutravidin beads.

For compound pull-down experiments, sepharose beads were derivatized with 0.1 mM VH125 bearing a free terminal amino group (NH_2_-VH125) essentially as described^36^.

Competition experiments were performed by spiking increasing concentration of compound or vehicle control into HeLa cell lysates and incubating for 45 min at 4 °C. Subsequently, beads were added and incubated for 2 h at 4 °C. After washing, bound proteins were eluted with SDS-sample buffer and prepared for tandem mass tags labelling and MS analysis as described^36^. Samples were dried in vacuo, resuspended in 0.1% formic acid in water and injected into a Dionex Ultimate 3000 UHPLC coupled to Orbitrap mass spectrometers (Thermo Fisher Scientific). Mascot 2.4.1 (Matrix Science) was used for protein identification, using 10 ppm mass tolerance for peptide precursors and 20 mDa (HCD) mass tolerance for fragment ions. Protein identification and data processing for quantification were performed as described^36^. Aliquots of the eluates from the pull-downs experiments were used for western blot analysis and anti-VHL mouse antibodies (BD Pharmingen #556347) used for detection.

### Cellular thermal shift assay

HeLa cells were harvested with trypsin/EDTA, suspended in PBS supplemented with protease inhibitor cocktail (Thermo Scientific) and lysed by four freeze-thaw cycles in dry ice/ethanol bath. The soluble fraction (lysate) was separated from the cell debris by centrifugation at 17,000*g* for 20 min at 4 °C. The lysate was divided into two aliquots, with one aliquot being treated with 100 μM of VH298 or 100 μM of FG-4592 and the other aliquot with vehicle (1% DMSO). After 10-30 min incubation at room temperature, the two fractions were divided into smaller aliquots (50 μl) and heated individually at different temperatures for 3 min followed by cooling for 3 min at room temperature. The heated lysates were centrifuged at 17,000 × *g* for 20 min at 4 °C, and the soluble fractions were analysed by SDS-PAGE and immunoblotted for anti-VHL (Cell Signaling Techonology) and anti-PHD2 (Bethyl Laboratories). Raw images were analysed using ImageJ. Data quantified relative to control samples incubated at the lowest temperature (40.4 °C).

### Co-immunonoprecipitation

HeLa cells were lysed in 1% Triton X-100, 20 mM Tris (pH 7.5), 150 mM NaCl, and one tablet of EDTA-free complete protease inhibitor mix (Roche) per 10 ml buffer. 300 μg of cell lysates were used per immunoprecipitation condition. Protein lysates were incubated with 1 μg of HIF-1α-OH antibody (Hydroxy-HIF-1α (Pro564), Cell Signaling Techonology) or 3 μg of rabbit IgG control antibody (Sigma) in a rotating platform at 4 °C overnight. 20 μl of packed protein-G-sepharose beads (Pierce) were used to recover the immuno-complexes, by incubation in a rotating platform for 1.5 h at 4 °C. Lysates were collected as flow through (FT) before 5 washes with 0.1% Tween-20 in 1 × PBS buffer. The complexes were eluted from beads with SDS loading buffer and resolved as described above by immunoblotting.

### Parallel artificial membrane permeability assay (PAMPA)

PAMPA was performed using a 96-well pre-coated BD Gentest PAMPA plate (BD Biosciences, UK). Each well was divided into two chambers: donor and acceptor, separated by a lipid-oil-lipid tri-layer constructed in a porous filter. The effective permeability, *P*_e_, of the compound was measured at pH 7.4. Stock solutions (5 mM) of the compound were prepared in DMSO. The compound was then further diluted to 10 μM in PBS, pH 7.4. The final DMSO concentration did not exceed 5% v/v. The compound dissolved in PBS was then added to the donor side of the membrane and PBS without compound was added to the acceptor side. The PAMPA plate was left at room temperature for 5 h, after which time, an aliquot (100 μl) was removed from both acceptor and donor compartments and mixed with acetonitrile (80 μl) containing an internal standard. The samples were centrifuged (10 min, 5 °C, 3270 × *g*) to sediment precipitated protein and sealed before ultra-performance liquid chromatography tandem mass-spectrometry (UPLC-MS/MS) analysis using a Quattro Premier XE (Waters Corp, USA). *P*_e_ was calculated as shown in the below equation:


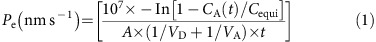


Where:

*C*_A_(*t*)= peak area of compound present in acceptor well at time *t*=18,000 s

*C*_equi_=[*C*_D_(*t*) × *V*_D_+*C*_A_(*t*) × *V*_A_]/(*V*_D_+*V*_A_)

*C*_D_(t)=peak area of compound present in donor well at time *t*=18,000 s

*A*=filter area

*V*_D_=donor well volume

*V*_A_=acceptor well volume

*t*=incubation time

Recovery of compound from donor and acceptor wells was calculated and data was only accepted when recovery exceeded 70%.

### CHIlogD_7.4_ measurement

The CHIlogD (chromatographic hydrophobicity index logD) at pH 7.4 was determined using retention time measurements on a HPLC Dionex system (Thermo Fisher) with a Luna C18 column (Phenomenex). Test samples in DMSO (10 mM) were diluted to a concentration of 0.25 mM using 50:50 acetonitrile:water. Mobile phase A was 10 mM ammonium acetate solution (pH 7.4) and mobile phase B was acetonitrile. HPLC method was as follows: 1 ml min^−1^ flow, temperature 20°C, injection volume 10 μl, gradient: 0 to 10.5 min 100% A, 10.5 to 14 min 100% B, 14 to 15 min 100% A. A calibration line was generated using a test mix of compounds (paracetamol, theophylline, caffeine, benzimidiazole, colchicine, carbamazepine, indole, propiophenone, butyrophenone, valerophenone and heptanophenone). The CHIlog*D* was calculated as previously described^78^,^79^.

### Cytotoxicity assay for mammalian cells

Cell viability was analysed using CellTiter-Glo Luminescent Cell Viability Assay (Promega). Cells were plated in 384-well plates (1,000 RCC4 cells per well and 1,500 HFF, HeLa or U2OS cells per well) one day prior and treated with VHL inhibitors and respective non-binding *cis-*analogues for 24 h. Cells were incubated with CellTiter-Glo reagent according to the manufacturer's protocol and luminescence was measured using PHERASTAR machine.

### Cytotoxicity assay for CTLs

Death of CTLs was analysed by staining with 4′,6-diamidino-2-phenylindole (DAPI). Cells were plated in 96-well plates at 1 × 10^6^ and treated with VHL inhibitors and respective non-binding *cis-*analogues for 24 h. Next, cells were spun down and re-suspended in HBSS containing DAPI to identify dead and dying populations. Data were acquired on FACSVerse machine (Becton Dickinson) and analysed using FlowJo software (TreeStar).

### Luciferase assay

U2OS cells stably express HRE-luciferase reporter were treated with indicated compounds in fresh medium for 32 h. Cells were lysed in passive lysis buffer (Promega), and luciferase assays were performed according to the manufacturer's instructions (Promega) and activity was measured using Berthold Lumat LB 9507 Luminometer. Results were normalized for protein concentration with all experiments being performed a minimum of three times before calculating means and s.d.s.

### Quantitative real-time PCR

Mammalian cells: RNA was extracted using the RNeasy Mini Kit (Qiagen) according to manufacturer's protocol. RNA was reverse transcribed using the iScript cDNA Synthesis kit (Bio-Rad). Real-time PCR was performed in triplicates using PerfeCTa SYBR Green FastMix (Quanta Biosciences) in C1000 Touch Thermal Cycler (Bio-Rad Laboratories). mRNA levels were calculated based on averaged CT values and normalized to β-actin mRNA levels. CTLs: RNA was extracted using the RNeasy Mini Kit (Qiagen) according to manufacturer's protocol. RNA was reverse transcribed using the qScript cDNA synthesis kit (Quanta). Real time PCR was performed in triplicates using SYBR Premix Ex Taq II (2 × ) (TaKaRa) in C1000 Thermal Cycler (Bio-Rad Laboratories). mRNA levels were calculated based on averaged CT values and normalized to TATA-binding protein (TBP) mRNA levels.

Primer sequences are available in the Supplementary Methods of the Supporting Information.

### Data availability

Data supporting the findings of this study are available within the article (and its Supplementary Information files) and from the corresponding author on reasonable request. Coordinates and structure factors have been deposited in the Protein Data Bank (PDB) with the accession code 5LLI.

## Additional information

**How to cite this article**: Frost, J. *et al*. Potent and selective chemical probe of hypoxic signalling downstream of HIF-α hydroxylation via VHL inhibition. *Nat. Commun.* **7**, 13312 doi: 10.1038/ncomms13312 (2016).

**Publisher's note:** Springer Nature remains neutral with regard to jurisdictional claims in published maps and institutional affiliations.

## Supplementary Material

Supplementary Information Supplementary Figures 1-11, Supplementary Tables 1-3, Supplementary Methods and Supplementary References.

Supplementary Data 1 Chemoproteomic target profiling of VHL inhibitors using a VH125- derived bead matrix using either VH125 or an inactive Pro-containing compound as competitor. Quantified proteins and IC50 values derived from reporter ion intensities are listed. Data are represented in Supplementary Figures 4 and 5, respectively.

Supplementary Data 2 Mass spectrometric quantification of proteins captured by immobilized HIF-1α NODD and CODD Hyp-modified peptides compared to unmodified peptides (control). Fold change (and Log2 fold change) values derived from reporter ion intensities are listed. Data are represented in Supplementary Figure 6a and 6b, respectively.

## Figures and Tables

**Table 1:**
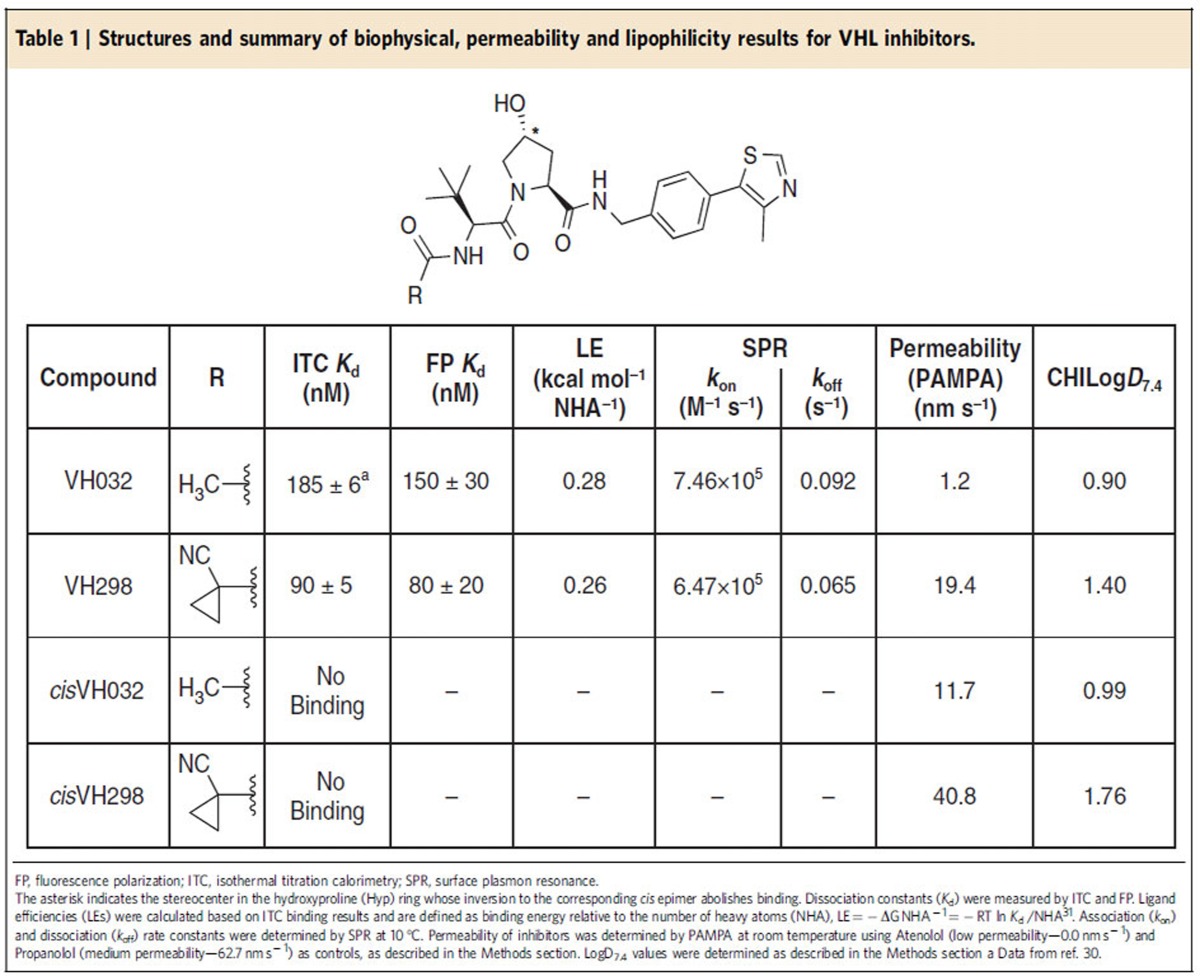
Structures and summary of biophysical, permeability and lipophilicity results for VHL inhibitors.

*Reaction conditions:**1**/LiHMDS/**2**/[Pd(*η*^3^-C_3_H_5_)Cl]_2_/S-IPr·HCl=200/200/100/2.5/5; 0.1 M of ketone **1**; T=30^o^C; B/L and *dr* was determined by ^1^H NMR, *dr* is the ratio of (±)-(*syn,anti*)-**3**/other diastereoisomers; Isolated yield. †T=50 ^o^C. ‡Solvent=THF. §OBoc of **2** was replaced with OP(OEt)_2_. ||The yield was determined by ^1^H NMR.
