# Supplementary material for: Potent and selective chemical probe of hypoxic signalling downstream of HIF-α hydroxylation via VHL inhibition
Source: Nat Commun. 2016 Nov 4;7:13312. doi: 10.1038/ncomms13312 (PMC5097156; doi:10.1038/ncomms13312)
Supplement: Supplementary Information — Supplementary Figures 1-11, Supplementary Tables 1-3, Supplementary Methods and Supplementary References. [file ncomms13312-s1.pdf]

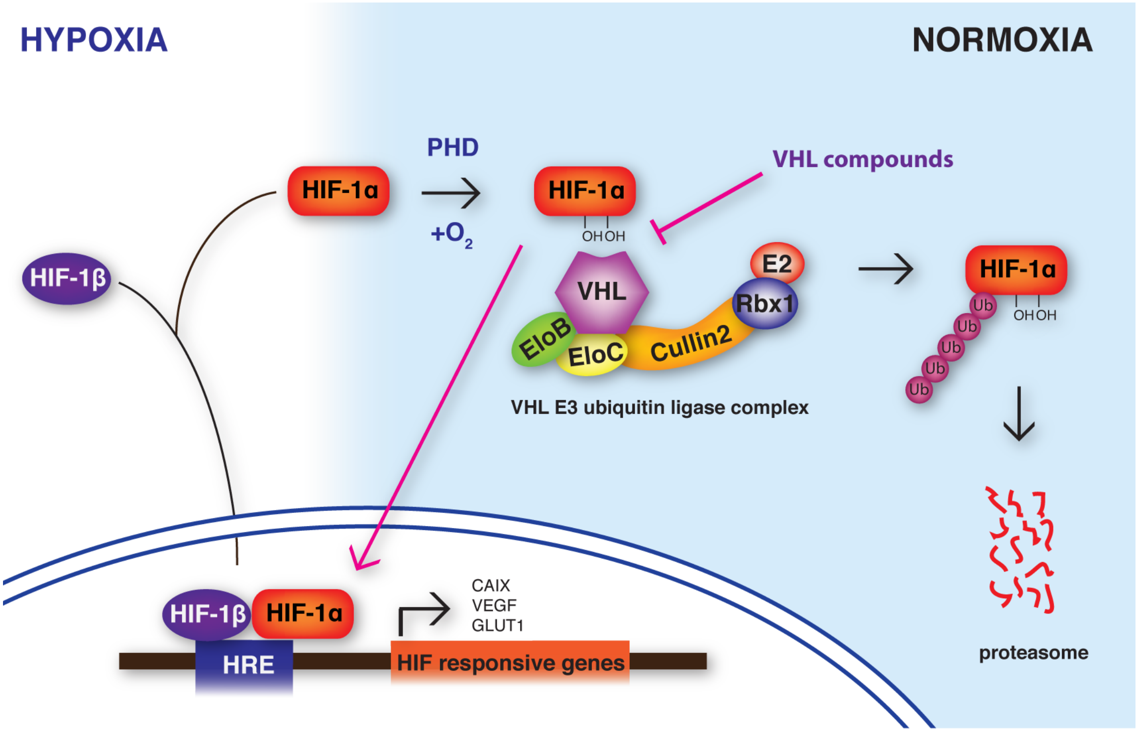

Supplementary Figure 1. Oxygen sensing and hypoxic signalling pathway. Under normoxic conditions, HIF-1α is hydroxylated, recognised by the $CRL2^{VHL}$ complex, ubiquitinated, and degraded by the proteasome. Under hypoxic conditions, HIF-1α dimerizes with HIF-1β and forms a transcriptionally active complex that binds to Hypoxia-Response Elements (HREs), thereby promoting a hypoxic response.

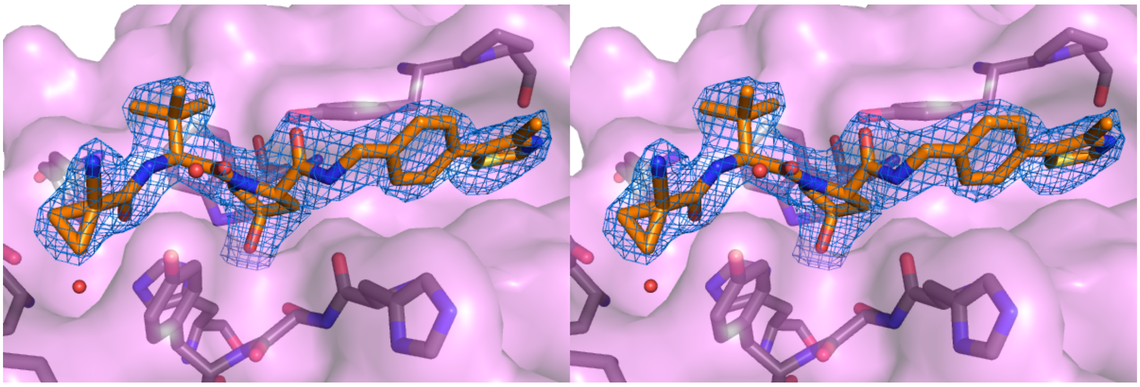

Supplementary Figure 2. Stereo view of the crystal structure of VH298 bound to VHL. $2F_o–F_c$ electron density (blue mesh) for the atoms around VH298 is shown to 1.5σ with a carve radius of 1.6 Å. VH298 is shown as sticks with orange carbons, and VHL is shown as violet surface and grey sticks.

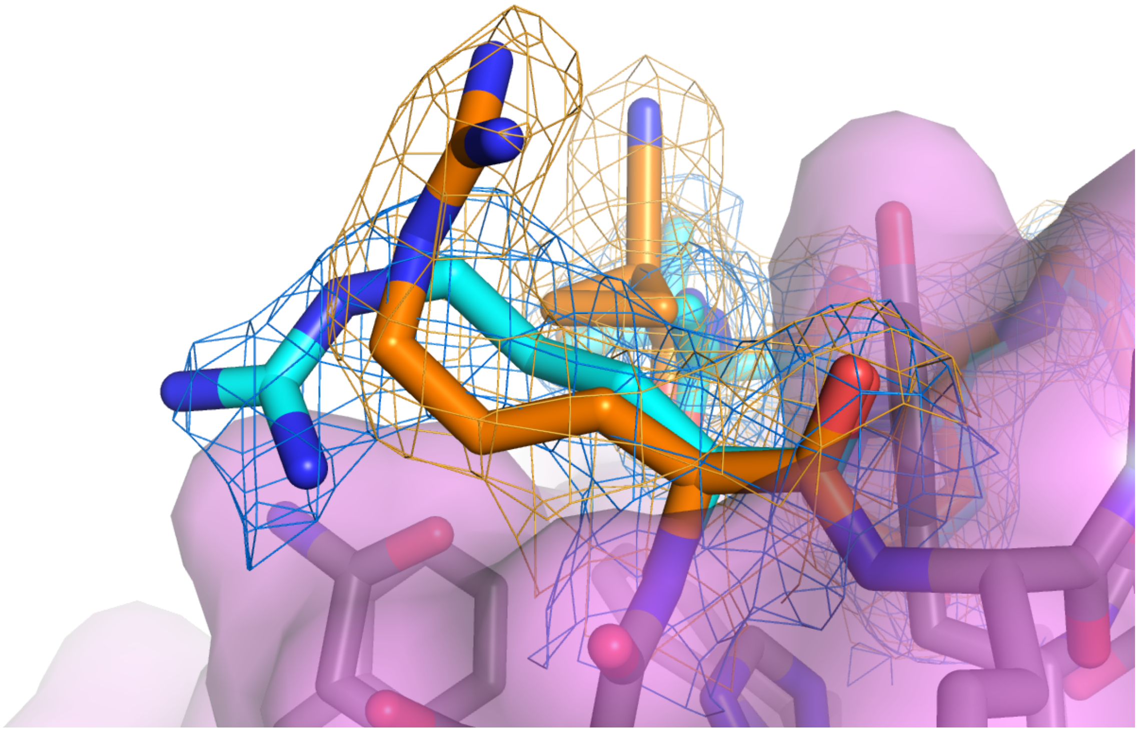

Supplementary Figure 3. Illustration of conformational differences in Arg69. Overlay of crystal structures of VHL (violet surface, residues shown as sticks with grey carbons) bound to VH032 (cyan sticks) and VH298 (orange sticks). The Arg69 side chain as well as the $2F_o–F_c$ electron density (mesh, contoured to 1σ with a carve radius of 1.6 Å) for each structure are shown in the corresponding colours.

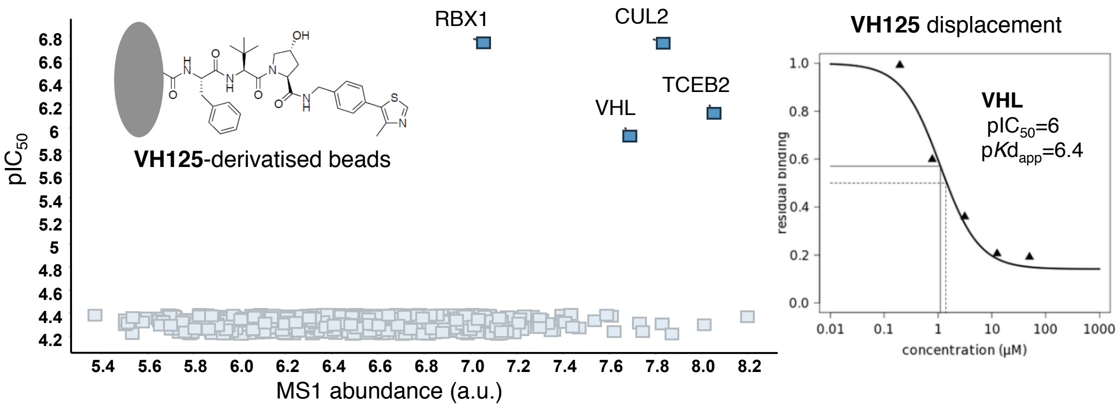

Supplementary Figure 4. Chemoproteomic competition binding experiment. Sepharose bead matrix was derivatized with a linkable VHL inhibitor (VH125[1]), an analog of VH298. Free VH125 compound was spiked into cell extracts over a range of concentrations. Of all proteins captured by the affinity matrix (see Supplementary Data 1), only the component subunits of the endogenous $CRL2^{VHL}$ complex (blue squares) showed dose-dependent competition (VHL data plotted in the right inset panel). X-axis indicates abundance of captured proteins inferred from signal intensities in the mass spectrometer (TOP3 method)[2]; on the y-axis binding potencies are plotted (p: -log10).

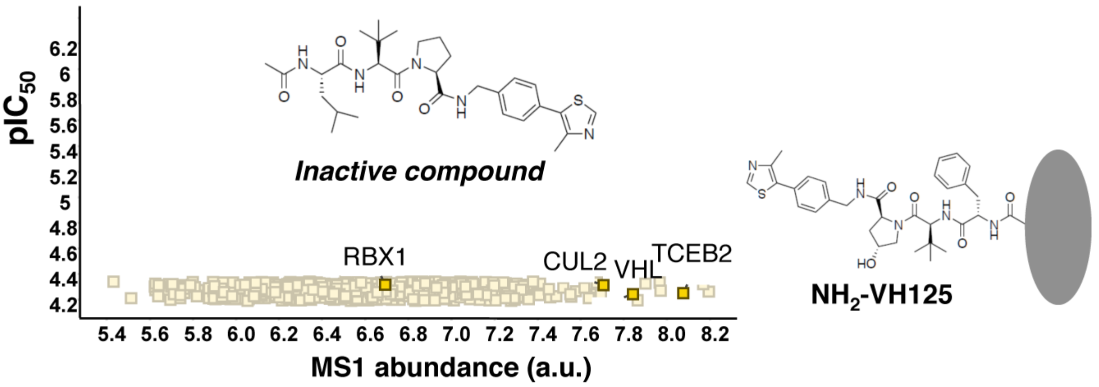

Supplementary Figure 5. Target specificity of VHL inhibitors. Chemoproteomic competition binding experiment using a sepharose-bound analog of VH125 as bead-matrix (NH_2-VH125), as shown in Supplementary Figure 4. Components of the $CRL2^{VHL}$ complex (yellow squares) are not displaced using an inactive compound bearing a proline (Pro) instead of a hydroxyproline (Hyp) as competitor.

a

b

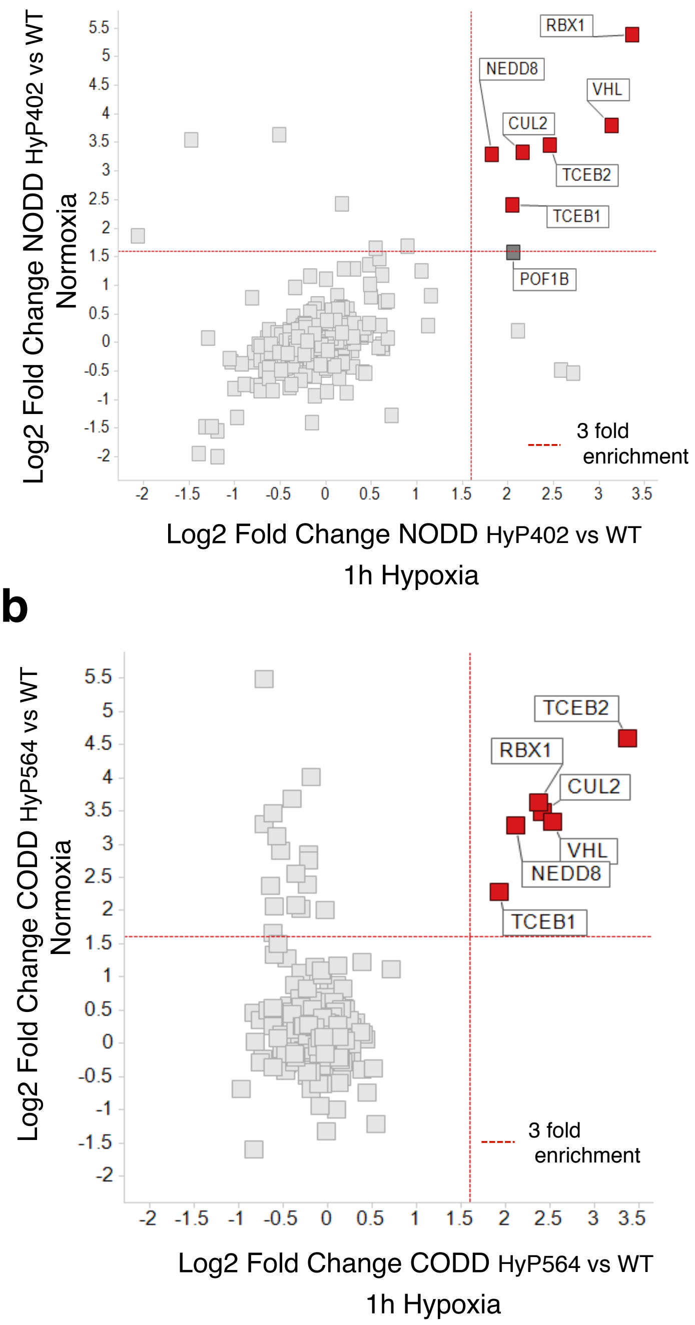

Supplementary Figure 6. CRL2^{VHL} complex selectively binds to immobilized HIF-1α peptides. Quantitative mass spectrometric analysis of proteins captured by immobilized HIF-1α N-terminal oxygen-degradation domain (NODD, a) and C-terminal oxygen-degradation domain (CODD, b) Hyp-modified peptides compared to unmodified peptides containing a Pro residue at the corresponding position. Experiments were performed using extracts generated from HeLa cells grown in normoxia or for 1 hr in hypoxia (1% O_2). Squares indicate identified proteins (see Supplementary Data 2); components of the CRL2^{VHL} complex are depicted in red. Red lines indicate 3-fold enrichment.

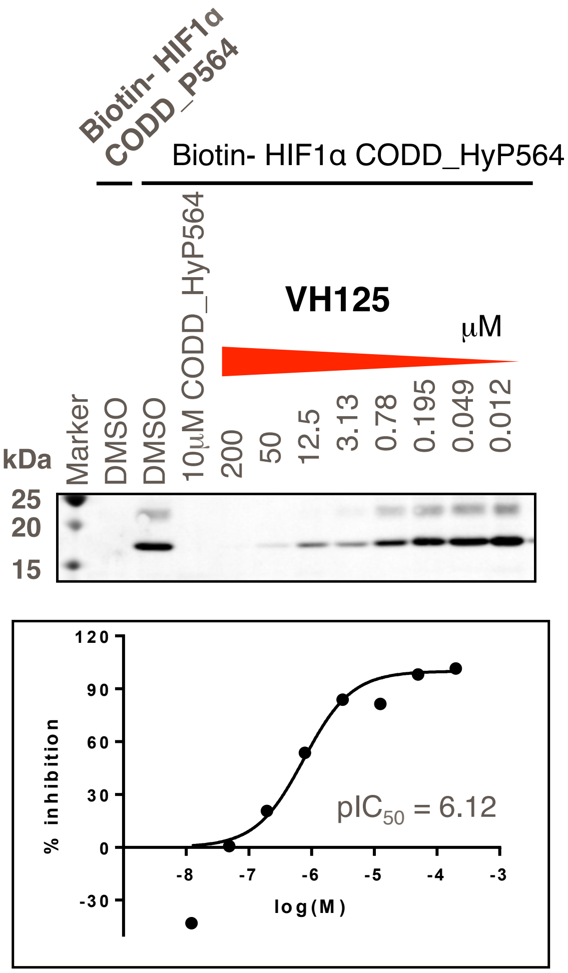

Supplementary Figure 7. VHL inhibitors effectively displace the native CRL2^{VHL} complex immobilized on HIF-1α peptides derivatized beads. Competition binding assay reveals concentration-dependent reduction of affinity-captured VHL protein on the immobilized HIF-1α CODD Hyp-modified peptide by the VHL inhibitor. Captured proteins were eluted with SDS buffer, immunostained using a VHL-specific antibody, and quantified (lower panel).

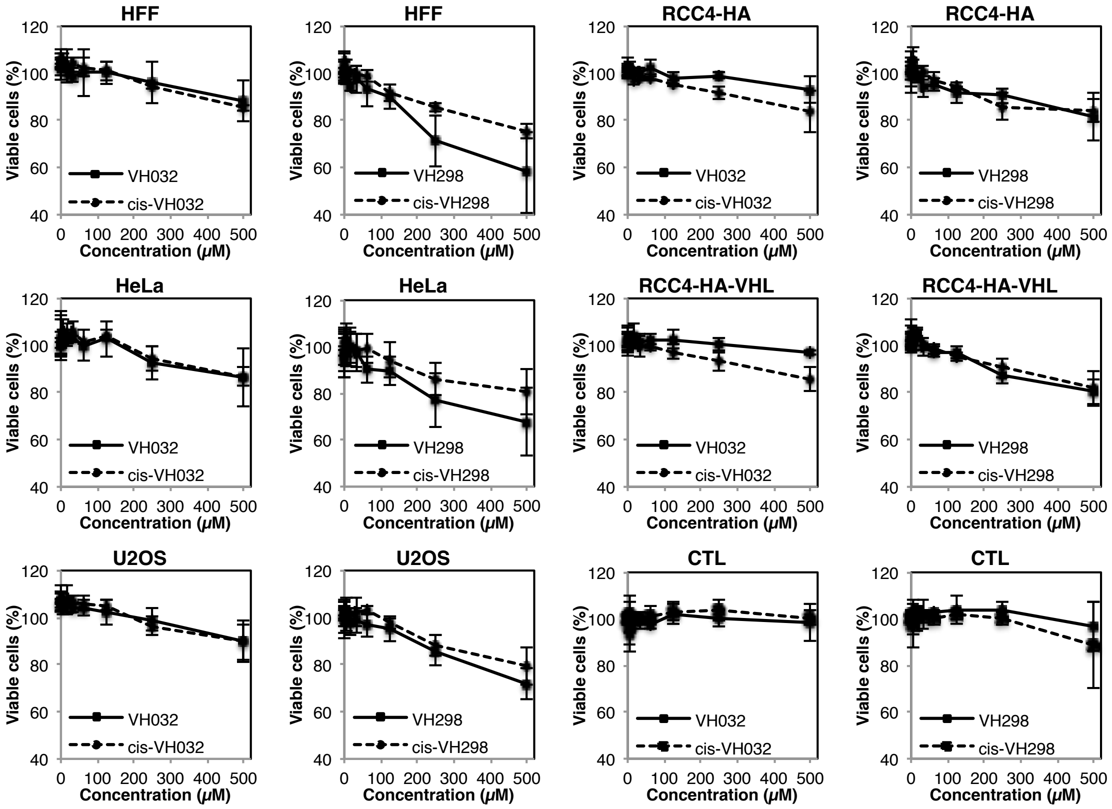

Supplementary Figure 8. VH032 and VH298 are not cytotoxic even at high micromolar concentrations. The viability of HFF, HeLa, U2OS, RCC4-HA and RCC4-HA-VHL cells treated with VH032, VH298, *cis*VH032 or *cis*VH298 for 24 h was determined using the CellTiter-Glo Luminescent Cell Viability Assay kit. The viability of CTLs was determined using DAPI staining and quantified by flow cytometry. Graphs depict the mean + SD of three independent biological replicates. Student's t-test was performed to calculate p values, and levels of significance are denoted as follows: **P*<0.05, ***P*<0.01 and, ****P*<0.001.

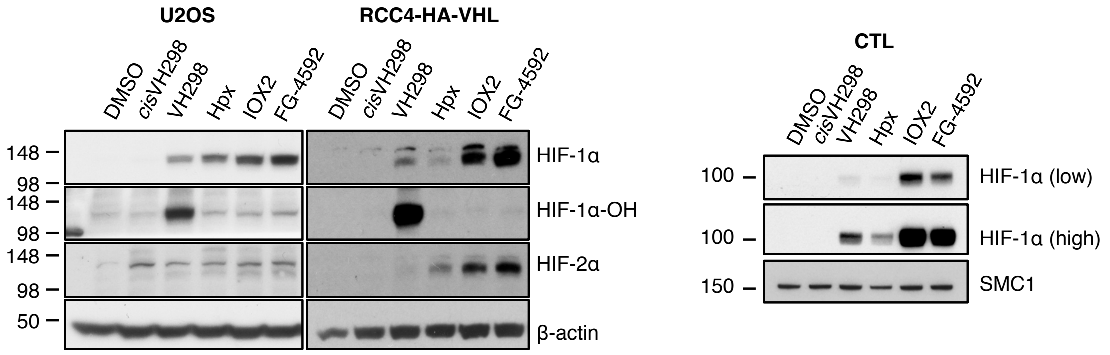

Supplementary Figure 9. Comparison of the effect of VH298, hypoxia and PHD inhibition in different cell lines (related to Figure 2b). Treatment of 1% DMSO, hypoxia (1% O_2), and 100 μM of indicated compounds in U2OS, RCC4 expressing HA-tagged VHL and CTL cells for 2 h, with the exception of 150 μM VH298 and *cis*VH298 in RCC4-HA-VHL. Protein levels were analyzed by immunoblotting using antibodies against the indicated proteins, with β-actin (Smc1 for CTLs) as the loading control. The blots shown are representative of three independent experiments.

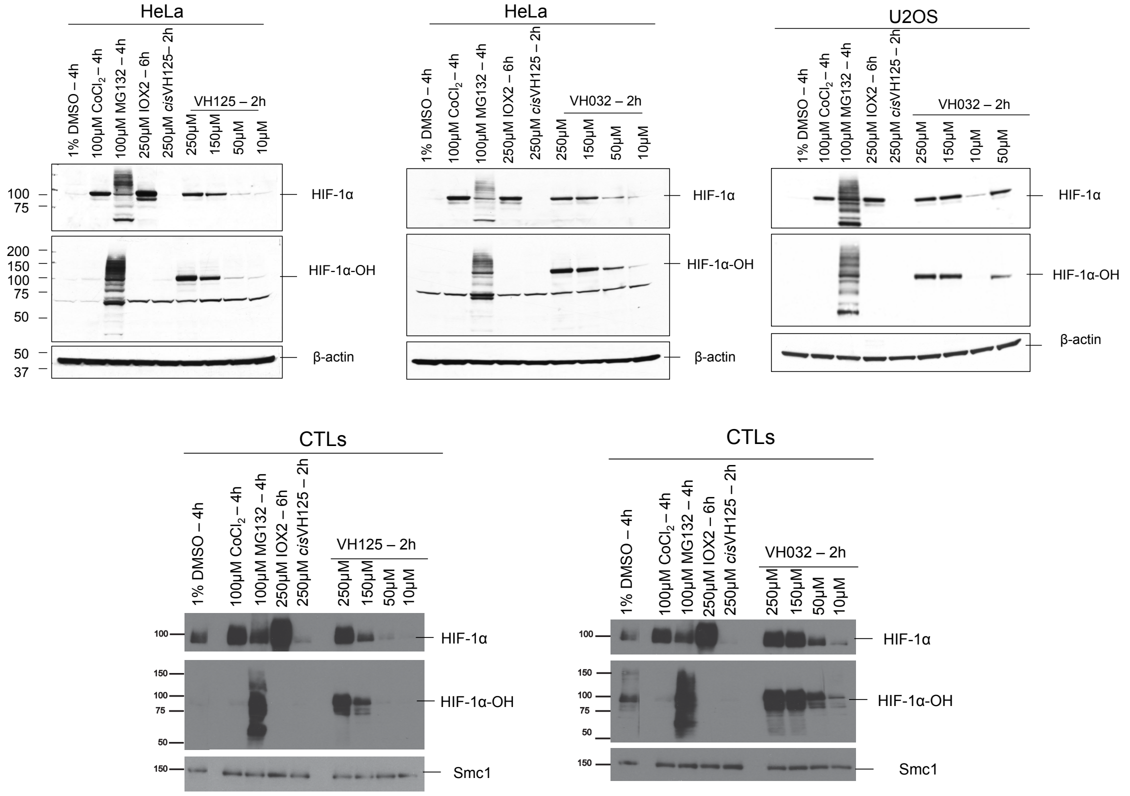

Supplementary Figure 10. VH125 and VH032 stabilize hydroxylated HIF-1α in HeLa, U2OS cells and CTLs. Western blots were performed on cell lysates treated with increasing concentrations of VH125 and VH032 and 250 μM of *cis*VH125 for 2 h. Cells were also subjected to normoxia for 4 h (1% DMSO), 100 μM $CoCl_2$ for 4 h, 100 μM MG132 for 4 h and 250 μM IOX2 for 6 h as controls.

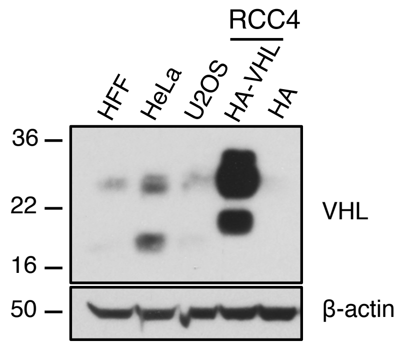

Supplementary Figure 11. HFF, HeLa, U2OS, RCC4 expressing HA tag or HA-tagged VHL were analyzed for VHL levels by western blotting.

Supplementary Table 1.
Crystallographic data processing and refinement statistics.

Values in parentheses are for the highest resolution shell.

**Synchrotron**	Diamond Light Source
Beamline	I04-1
Wavelength (Å)	0.9282
**Processing statistics**	
Space group	$P4_122$
Unit cell parameters (Å)	$a = b = 94.6$, $c = 368.6$
Resolution limits (Å)	49.6–2.40 (2.46–2.40)
Total reflections	440558 (3050)
Unique reflections	66540 (4365)
Completeness (%)	99.6 (99.5)
Multiplicity	6.6 (7.0)
R_{merge} (%)	9.9 (85.0)
$I/\sigma(I)$	12.5 (2.3)
$CC_{1/2}$ (%)	99.9 (80.4)
Wilson B factor (Å^2)	33.3
Mosaicity (°)	0.07
**Refinement statistics**	
Resolution limits (Å)	94.6–2.40 (2.46–2.40)
R_{work} (%)	19.6 (26.0)
R_{free} (%)	25.2 (34.1)
No. reflections	63122 (4531)
No. test reflections	3358 (252)
Model atoms	11505
Protein B factor (Å^2)	25.4
Ligand B factor (Å^2)	40.1
r.m.s.d. bonds (Å)	0.009
r.m.s.d. angles (°)	1.33
**Ramachandran plot**	
Favoured (%)	96.9
Allowed (%)	3.0
Disallowed (%)	0.1
**PDB code**	5LLI

Supplementary Table 2
VH298 kinase screening results

Kinase	Mean % of remaining kinase activity (±sd)	Kinase	Mean % of remaining kinase activity (±sd)
JNK1	119 (11)	PLK1	100 (2)
CK2	116 (6)	HIPK2	100 (2)
EF2K	116 (6)	TrkA	100 (12)
IRAK4	112 (9)	PKCa	99 (1)
RIPK2	112 (24)	CK1δ	99 (8)
Src	112 (3)	JAK3	99 (1)
TAK1	111 (25)	MST2	98 (5)
PDK1	110 (10)	PAK4	97 (5)
PRK2	110 (6)	TTK	97 (9)
TBK1	110 (5)	HER4	96 (5)
IGF-1R	110 (3)	MLK3	95 (6)
p38a MAPK	108 (5)	RSK1	94 (2)
PKD1	108 (1)	MARK3	94 (15)
GSK3b	107 (2)	CHK2	93 (7)
EPH-A2	107 (2)	PIM1	92 (2)
NEK6	106 (6)	S6K1	87 (1)
SmMLCK	105 (2)	MSK1	87 (6)
LKB1	105 (2)	MKK1	85 (3)
Lck	104 (2)	SGK1	83 (2)
SYK	103 (9)	CAMK1	81 (6)
VEG-FR	103 (8)	Aurora B	78 (4)
BTK	102 (4)	DYRK1A	76 (6)
CAMKKb	101 (12)	ROCK 2	75 (6)
AMPK (hum)	101 (18)	PKA	68 (2)
SRPK1	101 (1)	PKBa	54 (1)

VH298 was screened at 50 µM concentration against a panel of 50 kinases (Dundee MRC-PPU Express Screen, http://www.kinase-screen.mrc.ac.uk/services/express-screen). The remaining kinase activity was recorded in the end of the assay. The data is reported as average % activity remaining of assay duplicates for each kinase tested (standard deviation in brackets), ranked from highest to lowest.

Supplementary Table 3
VH298 receptor and ion-channels panel screening results.

Receptor	Reference compound	Mean % of control specific binding (±sd)
H2 (h)	cimetidine	140 (6)
CCK1 (CCKA) (h)	CCK-8s	127 (9)
A2A (h)	NECA	121 (8)
5-HT1B	serotonin	117 (0)
CB1 (h)	CP 55940	115 (10)
Ca2+ channel (L, verapamil site)	D 600	114 (7)
CCR1 (h)	MIP-1alpha	111 (1)
KV channel	alpha -dendrotoxin	111 (7)
BZD (central)	diazepam	106 (4)
Y1 (h)	NPY	106 (3)
mu (MOP) (h)	DAMGO	106 (4)
5-HT5a (h)	serotonin	105 (1)
NTS1 (NT1) (h)	neurotensin	103 (1)
D1 (h)	SCH 23390	103 (1)
sst (non-selective)	somatostatin-14	102 (5)
CXCR2 (IL-8B) (h)	IL-8	102 (5)
GABA (non-selective)	GABA	101 (8)
B2 (h)	NPC 567	101 (6)
H1 (h)	pyrilamine	101 (8)
alpha 1 (non-selective)	prazosin	99 (16)
5-HT7 (h)	serotonin	99 (4)
MC4 (h)	NDP-alpha -MSH	98 (5)
Y2 (h)	NPY	97 (5)
norepinephrine transporter (h)	protriptyline	96 (1)
EP4 (h)	PGE2	95 (2)
beta 2 (h)	ICI 118551	95 (2)
V1a (h)	[d(CH2)51,Tyr(Me)2]-AVP	95 (2)
M3 (h)	4-DAMP	95 (8)
M1 (h)	pirenzepine	94 (8)
M2 (h)	methoctramine	94 (3)
NK3 (h)	SB 222200	94 (4)
SKCa channel	apamin	93 (12)
NOP (ORL1) (h)	nociceptin	92 (2)
5-HT3 (h)	MDL 72222	92 (2)
5-HT6 (h)	serotonin	92 (4)
kappa (KOP)	U 50488	92 (2)
5-HT transporter (h)	imipramine	92 (2)
5-HT2B (h)	(±)DOI	91 (5)
D2S (h)	(+)butaclamol	91 (10)
A1 (h)	DPCPX	90 (7)
dopamine transporter (h)	BTCP	90 (5)

ETA (h)	endothelin-1	88 (5)
GAL2 (h)	galanin	87 (4)
alpha 2 (non-selective)	yohimbine	86 (2)
VPAC1 (VIP1) (h)	VIP	85 (15)
AT1 (h)	saralasin	84 (4)
5-HT2A (h)	ketanserin	83 (3)
beta 1 (h)	atenolol	83 (8)
5-HT1A (h)	8-OH-DPAT	82 (1)
Na+ channel (site 2)	veratridine	78 (5)
MT1 (ML1A) (h)	melatonin	75 (8)
NK2 (h)	[Nleu10]-NKA (4-10)	73 (12)
Cl- channel (GABA-gated)	picrotoxinin	69 (1)
delta (DOP) (h)	DPDPE	65 (3)
A3 (h)	IB-MECA	55 (7)

VH298 was screened at 50 µM concentration against a panel of receptor and ion channels (CEREP ExpresSprofile assay, http://www.cerep.fr/cerep/users/pages/downloads/Documents/Marketing/Pharmacology%20&%20ADME/Standard%20profiles/ExpresSProfile_2014v2LD.pdf). The remaining control specific binding of a reference compound was recorded in the end of the assay. The data is reported as average % of control specific binding of assay duplicates for each receptor tested (standard deviation in brackets), ranked from highest to lowest.

Supplementary Methods:

Synthesis of pVHL compounds

General Chemistry. Commercial available starting reagents for each reaction were purchased from Sigma Aldrich, Fluorochem, Apollo Scientific or Manchester Organics and used without further purification. All reactions were carried using anhydrous solvents. Analytical thin-layer chromatography (TLC) was performed on pre-coated TLC plates (layer 0.20 mm silica gel 60 with fluorescent indicator (UV 254: Merck). The TLC plates were air dried and revealed under UV lamp (254/365 nm). Flash column chromatography was performed used pre-packed silica gel cartridges (230-400 mesh, 40-63 mm; SiliCycle) using a Teledyne ISCO Combiflash Companion or Combiflash Retrieve using the solvent mixtures stated for each synthesis as mobile phase.

Liquid chromatography-mass spectrometry (LC-MS) analyses were performed with either an Agilent HPLC 1100 series connected to a Bruker Daltonics MicroTOF or an Agilent Technologies 1200 series HPLC connected to an Agilent Technologies 6130 quadrupole spectrometer or a Waters 2795 connected to a Waters ZQ Micromass spectrometer, where all instruments were connected to an diode array detector. All the final compounds used in all the experiments were evaluated after preparative LC-MS separations with a Waters X-bridge C18 column (50 mm x 2.1 mm x 3.5 mm particle size), with a mobile phase of water/acetonitrile+0.1% CHOOH or water/acetonitrile+0.1% NH_3, using a linear gradient from 80:20 to 5:95 over 3.5 min and then held for 1.5 min, at a flow rate of 0.5 mL/min. The purity of all the ligands was evaluated using the analytical LC-MS system described before and yield a purity >95%.

High-resolution electrospray measurements were performed on a Bruker Daltonics MicroTOF mass spectrometer. ^{1}H NMR and ^{13}C NMR spectra were recorded on a Bruker Avance II 500 spectrometer (^{1}H at 500.1 MHz; ^{13}C at 125.8 MHz) or on a Bruker DPX-400 Cryo spectrometer (^{1}H at 400.1 MHz; ^{13}C at 101 MHz). Chemical shifts (δ) are expressed in ppm reported using residual solvent as the internal reference in all cases. Signal splitting patterns are described as singlet (s), doublet (d), triplet (t), multiplet (m), or a combination thereof. Coupling constants (*J*) are quoted to the nearest 0.1 Hz.

The final compounds **VH032** and **VH125** were synthesized as described elsewhere[1].

General synthesis of (2*S*,4*R*)-1-((S)-2-(1-cyanocyclopropanecarboxamido)-3,3-dimethylbutanoyl)-4-hydroxy-N-(4-(4-methylthiazol-5-yl)benzyl)pyrrolidine-2-carboxamide, VH298

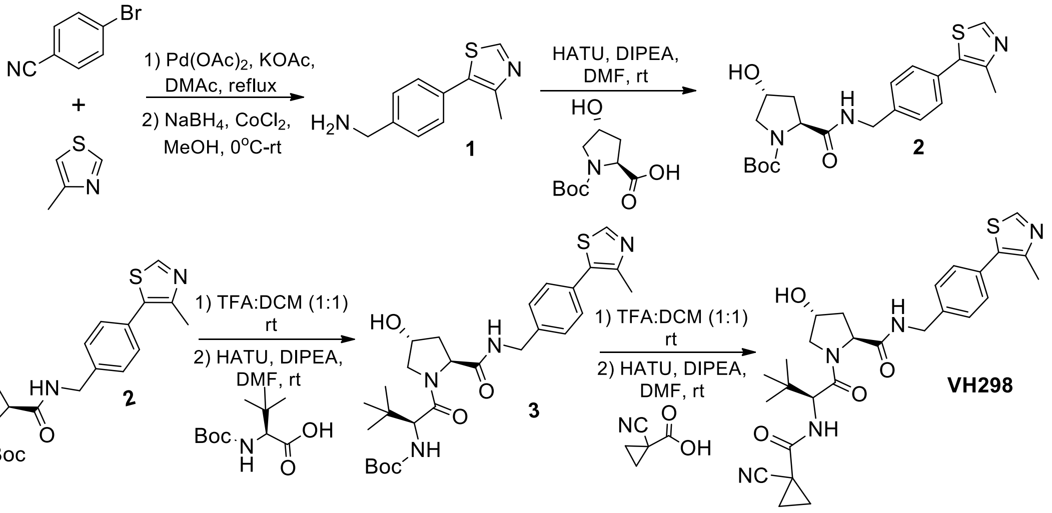

(4-(4-methylthiazol-5-yl)phenyl)methanamine, 1

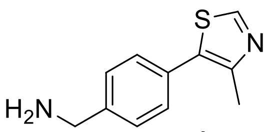

1

Synthesized as described previously[3].

(2*S*,4*R*)-*tert*-butyl 4-hydroxy-2-((4-(4-methylthiazol-5-yl)benzyl)carbamoyl)pyrrolidine-1-carboxylate, 2

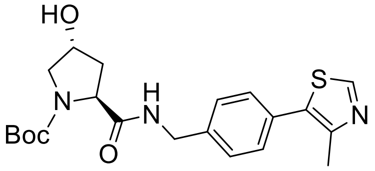

2

Synthesized as described previously[1].

***tert*-butyl((*S*)-1-((2*S*,4*R*)-4-hydroxy-2-((4-(4-methylthiazol-5-yl)benzyl)carbamoyl) pyrrolidin-1-yl)-3,3-dimethyl-1-oxobutan-2-yl)carbamate, 3**

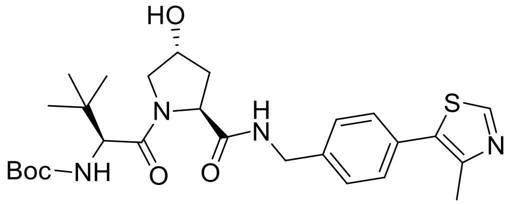

3

A solution of compound **2** (300 mg, 0.72 mmol) in 1:1 TFA:DCM (6 mL) was stirred at room temperature for 30 min. The mixture was evaporated under reduced pressure to give the corresponding deprotected intermediate (TFA salt) as brown oil without further purification (303 mg). To a solution of the deprotected intermediate from compound **2** (303 mg, 0.70 mmol, 1 equiv.) in DMF was added Boc-*L*-tertleucine (162 mg, 0.70 mmol, 1 equiv.). DIPEA (362 mg, 2.8 mmol, 4 equiv.) was added dropwise, and the mixture was stirred for 5 min at room temperature. HATU (293 mg, 0.77 mmol, 1.1 equiv.) was added and the mixture was stirred at room temperature for more 30 minutes. Water was added, and the mixture was extracted with ethyl acetate (3x). The combined organic phases were washed with brine (2x), dried over $MgSO_4$, and evaporated to afford the corresponding crude compound that was purified by flash column chromatography using a gradient of 10% to 70% acetone in heptane to yield the final compound **3** as a white solid (274 mg, 72%). ^{1}H NMR ($CDCl_3$, 500 MHz): δ 8.67 (s, 1H), 7.44 (t, 1H, *J* = 5.0), 7.34 (q, 4H, *J* = 15.0, 10.0 Hz), 5.18 (d, 1H, *J* = 10.0 Hz), 4.75 (t, 1H, *J* = 5.0 Hz), 4.58-4.51 (m, 2H), 4.31 (dd, 1H, *J* = 15.0, 5.0 Hz), 4.15 (d, 1H, *J* = 10 Hz), 4.06 (d, 1H, *J* = 10 Hz), 3.57 (dd, 1H, *J* = 10.0, 5.0 Hz), 2.58-2.51 (m, 4H), 2.13-2.09 (m, 1H) 1.40 (s, 9H), 0.91 (s, 9H); ^{13}C NMR ($CDCl_3$, 125 MHz): δ 172.8, 170.7, 156.6, 150.4, 148.6, 138.2, 131.7, 131.1, 129.7, 128.2, 80.6, 70.3, 59.0, 58.4, 56.6, 43.4, 35.8, 34.9, 28.4, 26.5, 16.2. HRMS (ESI) m/z: [M^++1] calculated for $C_{27}H_{39}N_4O_5S$: 531.2641; observed: 531.2653.

(2*S*,4*R*)-1-((S)-2-(1-cyanocyclopropanecarboxamido)-3,3-dimethylbutanoyl)-4-hydroxy-N-(4-(4-methylthiazol-5-yl)benzyl)pyrrolidine-2-carboxamid, VH298

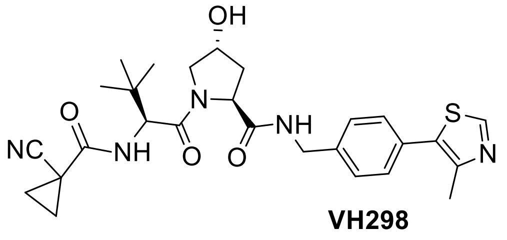

VH298

A solution of compound **3** (270 mg, 0.51 mmol) in 1:1 TFA:DCM (6 mL) was stirred at room temperature for 30 min. The mixture was evaporated under reduced pressure to give the corresponding deprotected intermediate (TFA salt) as brown oil without further purification (271 mg). To a solution of the deprotected intermediate from compound **3** (271 mg, 0.50 mmols, 1 equiv.) in DMF was added 1-cyanocyclopropanecarboxylic acid (56 mg, 0.50 mmol, 1 equiv.). DIPEA (257 mg, 2.0 mmol, 4 equiv.) was added dropwise, and the mixture was stirred for 5 min at room temperature. HATU (293 mg, 0.77 mmol, 1.1 equiv.) was added and the mixture was stirred at room temperature for 1 hour. Water was added, and the mixture was extracted with ethyl acetate (3x). The combined organic phases were washed with brine (2x), dried over $MgSO_4$, and evaporated to afford the corresponding crude compound that was purified by flash column chromatography using a gradient of 10% to 70% acetone in heptane to yield the final compound **VH298** as a white solid (128 mg, 48%). ^{1}H NMR ($CDCl_3$, 400 MHz): δ 8.68 (s, 1H), 7.36 (q, 4H, *J* = 16.0, 8.0 Hz), 6.96 (d, 1H, *J* = 8.0 Hz), 4.75 (t, 1H, *J* = 8.0 Hz), 4.61-4.56 (m, 2H), 4.42 (d, 1H, *J* = 8.0 Hz), 4.34 (dd, 1H, *J* = 16.0, 8.0 Hz), 3.95(d, 1H, *J* = 8.0 Hz), 3.62 (dd, 1H, *J* = 16.0, 8.0 Hz), 2.65-2.58 (m, 1H), 2.53 (s, 3H), 2.13-2.08 (m, 1H), 1.71-1.58 (m, 4H), 0.96 (s, 9H); ^{13}C NMR ($CDCl_3$, 101 MHz): δ 171.1, 170.4, 166.2, 150.5, 148.7, 138.0, 131.7, 131.3, 129.8, 128.4, 119.7, 70.4, 58.8, 58.5, 56.6, 43.5, 35.8, 35.2, 26.5, 18.1, 16.2, 13.8. HRMS (ESI) m/z: [M^++1] calculated for $C_{27}H_{34}N_5O_4S$: 524.2331; observed: 524.2344.

Synthesis of (2*S*,4*S*)-1-((S)-2-(1-cyanocyclopropanecarboxamido)-3,3-dimethylbutanoyl)-4-hydroxy-N-(4-(4-methylthiazol-5-yl)benzyl)pyrrolidine-2-carboxamide, *cis*VH298

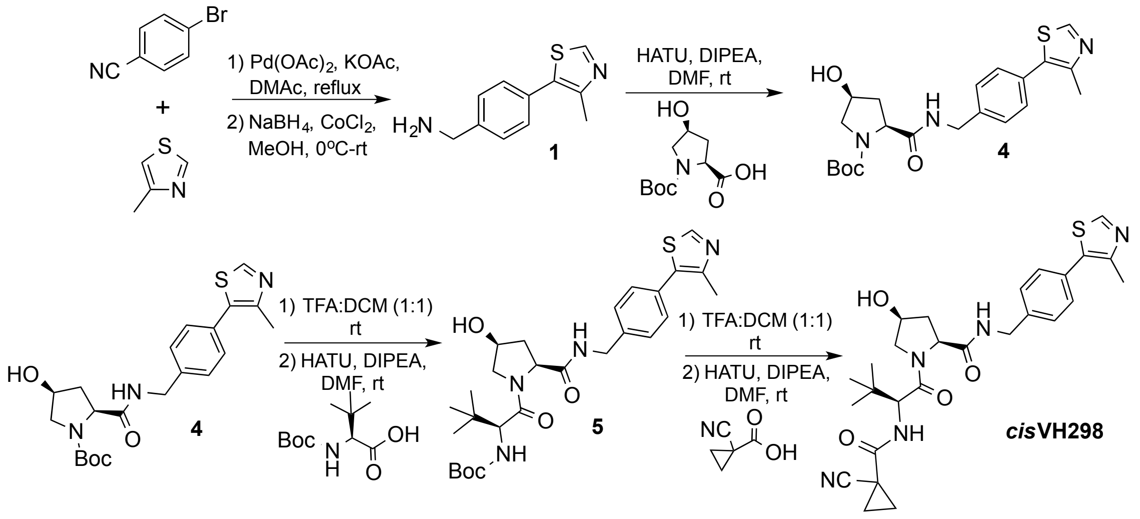

(2*S*,4*S*)-tert-butyl 4-hydroxy-2-((4-(4-methylthiazol-5-yl)benzyl)carbamoyl)pyrrolidine-1-carboxylate, 4

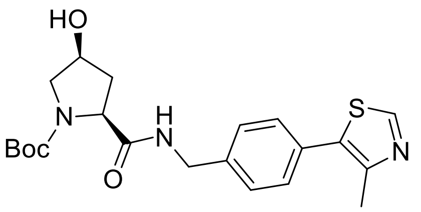

4

To a solution of compound **1** (500 mg, 2.43 mmol, 1 equiv.) in DMF was added added (2*S*,4*S*)-1-(tert-butoxycarbonyl)-4-hydroxypyrrolidine-2-carboxylic acid (565 mg, 2.43 mmol, 1 equiv.). DIPEA (1.26 g, 9.8 mmol, 4 equiv.) was added dropwise, and the mixture was stirred for 5 min at room temperature. HATU (1.02 g, 2.69 mmol, 1.1 equiv.) was added and the mixture was stirred at room temperature for more 30 minutes. Water was added, and the mixture was extracted with ethyl acetate (3x). The combined organic phases were washed with brine (2x), dried over $MgSO_4$, and evaporated to afford the corresponding crude compound that was purified by flash column chromatography using a gradient of 10% to 60% Acetone in Heptane to yield the final compound **4** as a white solid (587 mg, 58%). ^{1}H-NMR ($CDCl_3$, 400 MHz): δ 8.67 (s, 1 H), 7.54-7.51 (m, 1 H), 7.39-7.32 (m, 4 H), 4.58 (dd, 1 H, *J* = 14.9 , 7.1 Hz), 4.46-4.40 (m, 4H), 3.44-3.53 (m, 2H), 2.51 (s, 3H), 2.34-2.39 (m, 1 H), 2.14-2.23 (m, 1 H), 1.45 (s, 9H); ^{13}C-NMR ($CDCl_3$, 101 MHz): δ 173.5, 162.8, 155.9, 150.4, 148.7, 137.7, 131.2, 129.7, 127.8, 81.0, 70.9, 59.7, 57.2, 55.9, 35.9, 28.4, 16.2 ; HRMS (ESI) m/z: [M^++1] calculated for $C_{21}H_{28}N_3O_4S$ [M+1] 418.1800, found 418.1795.

***tert*-butyl ((S)-1-((2S,4S)-4-hydroxy-2-((4-(4-methylthiazol-5-yl)benzyl)carbamoyl) pyrrolidin-1-yl)-3,3-dimethyl-1-oxobutan-2-yl)carbamate, 5**

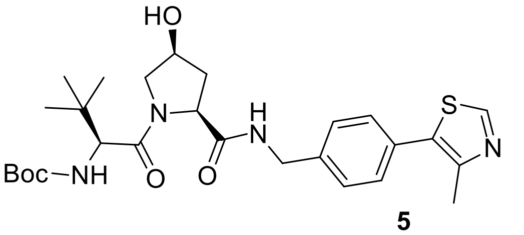

5

A solution of compound **4** (580 mg, 1.39 mmol) in 1:1 TFA:DCM (6 mL) was stirred at room temperature for 30 min. The mixture was evaporated under reduced pressure to give the corresponding deprotected intermediate (TFA salt) as brown oil without further purification (576 mg, 1.34 mmol, 98%). To a solution of the deprotected intermediate from compound **4** (576 mg, 1.34 mmol, 1 equiv.) in DMF was added Boc-*L*-tertleucine (309 mg, 1.34 mmol, 1

equiv.). DIPEA (690 mg, 5.3 mmol, 4 equiv.) was added dropwise, and the mixture was stirred for 5 min at room temperature. HATU (558 mg, 1.47 mmol, 1.1 equiv.) was added and the mixture was stirred at room temperature for more 30 minutes. Water was added, and the mixture was extracted with ethyl acetate (3x). The combined organic phases were washed with brine (2x), dried over $MgSO_4$, and evaporated to afford the corresponding crude compound that was purified by flash column chromatography using a gradient of 10% to 70% Acetone in Heptane to yield the final compound **5** as a white solid (531 mg, 72%). ^{1}H-NMR ($CDCl_3$, 400 MHz) δ 8.69 (s, 1H), 7.52-7.56 (m, 1H), 7.33-7.39 (m, 4H), 5.56 (s, 1H), 5.12 (d, 1H, *J*= 8.0 Hz), 4.77 (d, 1H, *J* = 8.0 Hz), 4.64 (dd, 1H, *J* = 16.0, 8.0 Hz), 4.48 (s, 1H), 4.29 (dd, 1H, *J* = 16.0, 8.0 Hz), 4.18 (d, 1H, *J* = 8.0 Hz), 3.78-3.91 (m, 2 H), 2.52 (s, 3H), 2.36-2.40 (m, 1H), 2.16-2.23 (m, 1H), 1.41 (s, 9 H), 0.90 (s, 9 H); ^{13}C-NMR ($CDCl_3$, 101 MHz) δ 173.0, 172.7, 155.8, 150.6, 148.6, 137.4, 131.7, 131.4, 129.8, 128.3, 80.0, 71.2, 60.0, 58.7, 58.5, 35.1, 32.0, 28.5, 26.4, 22.8, 16.1, 14.3; HRMS (ESI) m/z: [M^++1] calculated for $C_{27}H_{39}N_4O_5S$ [M+1] 531.2636, found 531.2660.

(2*S*,4*S*)-1-((S)-2-(1-cyanocyclopropanecarboxamido)-3,3-dimethylbutanoyl)-4-hydroxy-N-(4-(4-methylthiazol-5-yl)benzyl)pyrrolidine-2-carboxamide, *cis*VH298

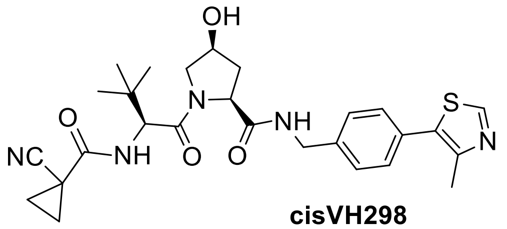

cisVH298

A solution of compound **5** (300 mg, 0.56 mmol) in 1:1 TFA:DCM (6 mL) was stirred at room temperature for 30 min. The mixture was evaporated under reduced pressure to give the corresponding deprotected intermediate (TFA salt) as brown oil without further purification (302 mg, 0.55 mmol, 98%).

To a solution of the deprotected intermediate from compound **5** (302 mg, 0.55 mmol, 1 equiv.) in DMF was added 1-cyanocyclopropanecarboxylic acid (61 mg, 0.55 mmol, 1 equiv.). DIPEA (287 mg, 2.2 mmol, 4 equiv.) was added dropwise, and the mixture was stirred for 5 min at room temperature. HATU (237 mg, 0.62 mmol, 1.1 equiv.) was added and the mixture was stirred at room temperature for 1 hour. Water was added, and the mixture was extracted with ethyl acetate (3x). The combined organic phases were washed with brine (2x), dried over $MgSO_4$, and evaporated to afford the corresponding crude compound that was purified by flash column chromatography using a gradient of 10% to 70% acetone in heptane to yield the final

compound ***cis*VH298** as a white solid (162 mg, 56%). ^{1}H NMR ($CDCl_3$, 400 MHz): δ 8.83 (s, 1H), 7.44-7.37 (m, 5H), 6.91 (d, 1H, J = 8.0 Hz) , 4.76 (d, 1H, J = 12.0 Hz), 4.67 (dd, 1H, J = 16.0, 8.0 Hz), 4.50-4.45 (m, 2H), 4.32 (dd, 1H, J = 16.0, 4.0 Hz), 3.85-375 (m, 2H), 2.55 (s, 3H), 2.38 (d, 1H, J = 12.0 Hz), 2.23-2.16 (m, 1H), 1.70-1.50 (m, 4H), 0.96 (s, 9H); ^{13}C NMR ($CDCl_3$, 101 MHz): δ 172.7, 171.4, 165.5, 151.7, 151.6, 133.2, 129.8, 128.6, 119.8, 71.2, 60.2, 58.7, 58.1, 43.7, 35.6, 35.2, 26.4, 18.0, 13.8. HRMS (ESI) m/z: [M^{+}+1] calculated for $C_{27}H_{34}N_5O_4S$: 524.2331; observed: 524.2329.

General synthesis of (2*S*,4*S*)-1-((S)-2-acetamido-3,3-dimethylbutanoyl)-4-hydroxy-N-(4-(4-methylthiazol-5-yl)benzyl)pyrrolidine-2-carboxamide, *cis*VH032

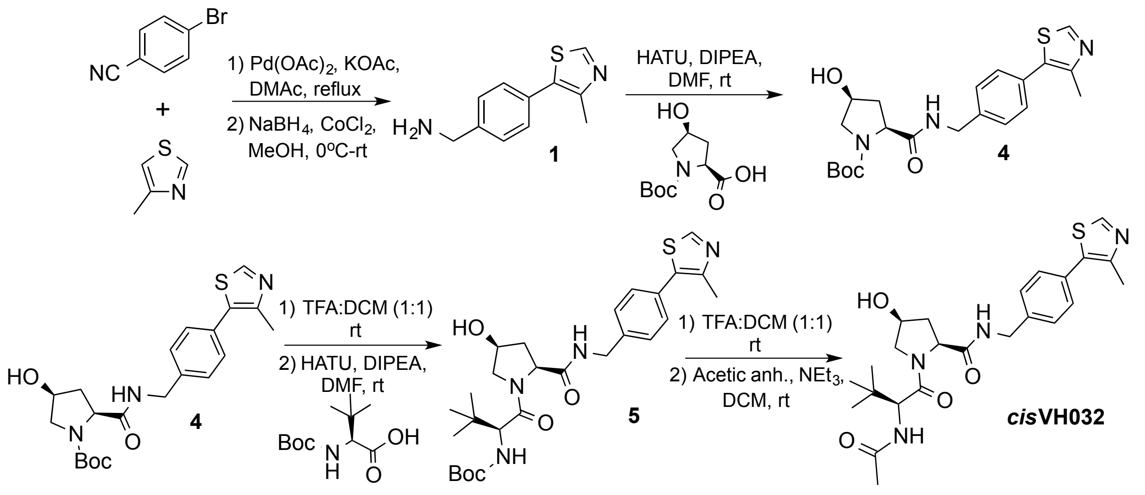

(2S,4S)-1-((S)-2-acetamido-3,3-dimethylbutanoyl)-4-hydroxy-N-(4-(4-methylthiazol-5-yl)benzyl)pyrrolidine-2-carboxamide, *cis*VH032

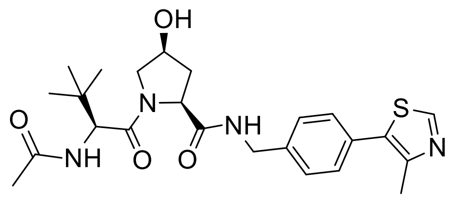

cisVH032

A solution of compound **5** (220 mg, 0.41 mmol) in 1:1 TFA:DCM (6 mL) was stirred at room temperature for 30 min. The mixture was evaporated under reduced pressure to give the corresponding deprotected intermediate (TFA salt) as brown oil without further purification (221 mg, 0.40 mmol, 98%). The deprotected intermediate from compound **5** (221 mg, 0.40

mmol, 1 equiv.) was dissolved in DCM, and triethylamine (123 mg, 1.20 mmol, 3 equiv) was added to the solution. After stirring the mixture for 10 min at room temperature, acetic anhydride (62 mg, 0.61 mmol, 1.5 equiv) was added and the reaction was stirred 90 min at room temperature. The solvents were evaporated under reduced pressure to give the corresponding crude, which was purified by flash column chromatography using a gradient of 10% to 70% Acetone in Heptane to yield the final compound ***cis*VH032** as white solid (124 mg, 65%). ^{1}H NMR ($CDCl_3$, 400 MHz): ^{1}H NMR ($CDCl_3$, 400 MHz): δ 8.71 (s, 1H), 7.37 (q, 4H, *J* = 18.0, 8.0 Hz), 5.97 (d, 1H, *J* = 8.0 Hz), 4.73 (d, 1H, *J* = 8 Hz), 4.66 (dd, 1H, *J* = 16.0, 8.0 Hz), 4.54-4.47 (m, 2H), 4.30 (dd, 1H, *J* = 16.0, 8.0 Hz), 3.92 (dd, 1H, *J* = 16.0, 8.0 Hz), 3.80 (d, 1H, *J* = 12.0 Hz), 2.52 (s, 3H), 2.38 (d, 1H, *J* = 12.0 Hz), 2.21-2.15 (m, 1H), 1.99 (s, 3H), 0.91 (s, 9H); ^{13}C NMR ($CDCl_3$, 101 MHz): δ 172.6, 172.5, 170.2, 150.6, 145.7, 137.4, 131.4, 129.8, 128.4, 71.2, 60.1, 58.8, 57.1, 43.7, 35.1, 26.4, 23.4, 16.2. HRMS (ESI) m/z: [M^++1] calculated for $C_{24}H_{33}N_4O_4S$: 473.2222; observed: 473.2198.

General synthesis of (2*S*,4*S*)-1-((S)-2-((S)-2-acetamido-3-phenylpropanamido)-3,3-dimethylbutanoyl)-4-hydroxy-N-(4-(4-methylthiazol-5-yl)benzyl)pyrrolidine-2-carboxamide, *cis*VH125

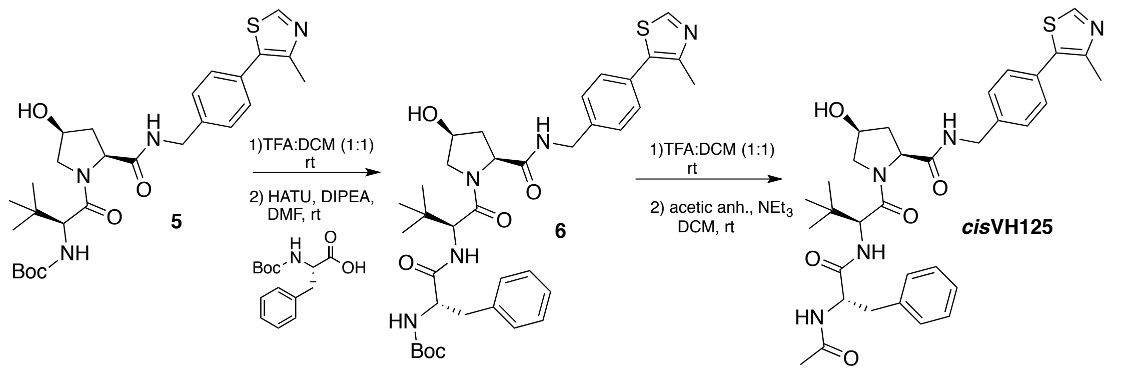

tert-butyl((S)-1-(((S)-1-((2S,4S)-4-hydroxy-2-((4-(4-methylthiazol-5-yl) benzyl) carbamoyl) pyrrolidin-1-yl)-3,3-dimethyl-1-oxobutan-2-yl)amino)-1-oxo-3-phenylpropan-2-yl) carbamates, 6

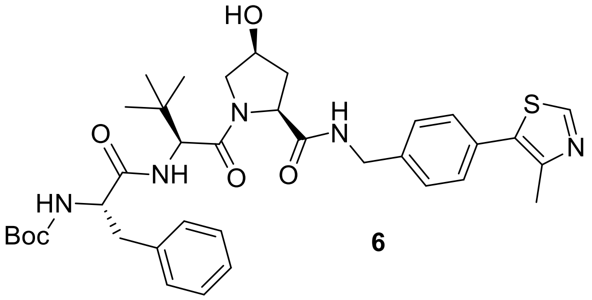

6

A solution of compound **5** (300 mg, 0.56 mmol) in 1:1 TFA:DCM (6 mL) was stirred at room temperature for 30 min. The mixture was evaporated under reduced pressure to give the corresponding deprotected intermediate (TFA salt) as brown oil without further purification (302 mg, 0.55 mmol, 98%).

To a solution of the deprotected intermediate from compound **5** (302 mg, 0.55 mmol, 1 equiv.) in DMF was added Boc-*L*-phenylalanine (146 mg, 0.55 mmol, 1 equiv.). DIPEA (287 mg, 2.20 mmol, 4 equiv.) was added dropwise, and the mixture was stirred for 5 min at room temperature. HATU (237 mg, 0.62 mmol, 1.1 equiv.) was added and the mixture was stirred at room temperature for more 30 min. Water was added, and the mixture was extracted with ethyl acetate (3x). The combined organic phases were washed with brine (2x), dried over $MgSO_4$, and evaporated to afford the corresponding crude compound that was purified by flash column chromatography using a gradient of 10% to 70% acetone in heptane to yield the final compound **6** as a lime solid (222 mg, 58%). ^{1}H NMR ($CDCl_3$, 400 MHz): δ 8.67 (s, 1H), 7.32 (q, 4H, *J* = 16.0, 8.0 Hz), 7.22-7.04 (m, 5H), 4.70-4.58 (m, 2H), 4.54-4.42 (m, 3H), 4.35 (dd, 1H, *J* = 15.0, 5.0 Hz), 3.95 (d, 1H, *J* = 10.0 Hz), 3.80 (dd, 1H, *J* = 11.0, 4.0 Hz), 2.90 (d, 2H, *J* = 5.0 Hz), 2.50 (s, 3H), 2.17-2.14 (m, 1H), 2.04-1.95 (m, 1H), 1.31 (s, 9H), 0.94 (s, 9H). ^{13}C NMR ($CDCl_3$, 101 MHz): δ 173.1, 171.6, 171.5, 155.8, 150.4, 148.6, 137.5, 137.0, 131.6, 131.2, 129.7, 129.3, 128.6, 128.1, 126.9, 80.4, 71.3, 59.8, 58.6, 57.2, 55.7, 43.6, 38.7, 36.1, 35.7, 28.3, 26.6, 16.2. HRMS (ESI) m/z: [M^++1] calculated for $C_{36}H_{48}N_5O_6S$: 678.3320; observed: 678.3332.

(2*S*,4*S*)-1-((S)-2-((S)-2-acetamido-3-phenylpropanamido)-3,3-dimethylbutanoyl)-4-hydroxy-N-(4-(4-methylthiazol-5-yl)benzyl)pyrrolidine-2-carboxamide (*cis*VH125)

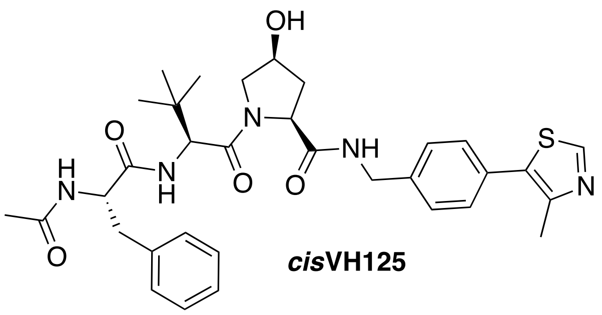

***cis*VH125**

A solution of compound **6** (220 mg, 0.32 mmol) in 1:1 TFA:DCM (6 mL) was stirred at room temperature for 30 min. The mixture was evaporated under reduced pressure to give the corresponding deprotected intermediate (TFA salt) as brown oil without further purification (219 mg, 0.32 mmol, 98%).

The deprotected intermediate from compound **6** (219 mg, 0.32 mmol, 1 equiv.) was dissolved in DCM, and triethylamine (96 mg, 0.95 mmol, 3 equiv) was added to the solution. After stirring the mixture for 10 min at room temperature, acetic anhydride (48 mg, 0.47 mmol, 1.5 equiv) was added and the reaction was stirred 90 min at room temperature. The solvents were evaporated under reduced pressure to give the corresponding crude, which was purified by flash column chromatography using a gradient of 10% to 70% Acetone in Heptane to yield the final compound ***cis*VH125** as white solid (113 mg, 56%). ^{1}H NMR ($CDCl_3$, 400 MHz): δ 8.68 (s, 1H), 7.86 (m, 1H), 7.34 (q, 4H, *J* = 18.0, 8.0 Hz), 7.21-7.13 (m, 5H), 7.06-7.04 (m, 2H), 4.79 (q, 1H, *J* = 15.0, 7.0 Hz), 4.66 (d, 1H, *J* = 9.0 Hz), 4.59-4.52 (m, 2H), 4.49-4.44 (m, 1H), 4.34 (dd, 1H, *J* = 15.0, 5.0 Hz), 3.91 (d, 1H, *J* = 12.0 Hz), 3.78 (dd, 1H, *J* = 11.0, 4.0 Hz), 3.48 (s, 2H), 2.93 (d, 2H, *J* = 7.0 Hz), 2.51 (s, 3H), 2.27 (d, 1H, *J* = 7.0 Hz), 2.15-2.08 (m, 1H), 1.90 (s, 3H), 0.92 (s, 9H). ^{13}C NMR ($CDCl_3$, 101 MHz): δ 172.9, 171.6, 170.9, 170.3, 150.5, 148.7, 137.4, 136.5, 131.6, 131.4, 129.7, 129.2, 128.6, 128.3, 127.1, 71.2, 59.8, 58.7, 57.4, 54.3, 51.0, 43.7, 38.5, 35.9, 26.6, 23.2, 16.2. HRMS (ESI) m/z: [M^++1] calculated for $C_{33}H_{42}N_5O_5S$: 620.2901; observed: 620.2906.

Primers for rt-PCR. Primer sequences used are listed.

Mammalian cells:
GLUT1 forward, 5' - TTCACTGTCGTGTCGCTGTTT -3'
GLUT1 reverse, 5' - AGCGCGATGGTCATGAGTAT -3'
CA9 forward, 5' - CGGAAGAAAACAGTGCCTATGAG -3'
CA9 reverse, 5'- CAGGGCGGTGTAGTCAGAGA -3'
EPO forward, 5'- GCTGAACACTGCAGCTTGAA -3'
EPO reverse, 5' - CAGACTTCTACGGCCTGCTG -3
β-actin forward, 5' - CTCTTCCAGCCTTCCTTCCTG -3'
β-actin reverse, 5'- GAAGCATTTGCGGTGGACGAT -3'

CTLs:
GLUT1 forward, CCAGCAGCAAGAAGGTGAC -3'
GLUT1 reverse, 5'-ATGTTTGATTGTAGAACTCCTC -3'
PHD2 forward, 5'-CTGTGGAACAGCCCTTTTTG -3'
PHD2 reverse, CGAGTCTCTCTGCGAATCCT -3'
TBP forward, 5'-GGGGAGCTGTGATGTGAA GT -3'
TBP reverse 5'- CCAGGAAATAATTCTGGCTCAT -3'

Supplementary References

1. Galdeano, C. *et al*. Structure-guided design and optimization of small molecules targeting the protein-protein interaction between the von Hippel-Lindau (VHL) E3 ubiquitin ligase and the hypoxia inducible factor (HIF) alpha subunit with in vitro nanomolar affinities. *J Med Chem* **57,** 8657–8663 (2014).
2. Grossmann, J. *et al*. Implementation and evaluation of relative and absolute quantification in shotgun proteomics with label-free methods. *J Proteomics* **73,** 1740–1746 (2010).
3. Buckley, D. L. *et al*. Small-Molecule Inhibitors of the Interaction between the E3 Ligase VHL and HIF1α. *Angew Chem Int Ed Engl* **51,** 11463–11467 (2012).
